# Genome of the pincer wasp *Gonatopus flavifemur* reveals unique venom evolution and a dual adaptation to parasitism and predation

**DOI:** 10.1186/s12915-021-01081-6

**Published:** 2021-07-27

**Authors:** Yi Yang, Xinhai Ye, Cong Dang, Yunshen Cao, Rui Hong, Yu H. Sun, Shan Xiao, Yang Mei, Le Xu, Qi Fang, Huamei Xiao, Fei Li, Gongyin Ye

**Affiliations:** 1 State Key Laboratory of Rice Biology and Ministry of Agricultural and Rural Affairs Key Laboratory of Molecular Biology of Crop Pathogens and Insects, Zhejiang University, Hangzhou, China; 2 Department of Biology, University of Rochester, Rochester, NY USA; 3 Key Laboratory of Crop Growth and Development Regulation of Jiangxi Province, College of Life Sciences and Resource Environment, Yichun University, Yichun, China

**Keywords:** Parasitoid wasp, Dryinidae, Genome size, Predation, Genome sequencing, Venom

## Abstract

**Background:**

Hymenoptera comprise extremely diverse insect species with extensive variation in their life histories. The Dryinidae, a family of solitary wasps of Hymenoptera, have evolved innovations that allow them to hunt using venom and a pair of chelae developed from the fore legs that can grasp prey. Dryinidae larvae are also parasitoids of Auchenorrhyncha, a group including common pests such as planthoppers and leafhoppers. Both of these traits make them effective and valuable for pest control, but little is yet known about the genetic basis of its dual adaptation to parasitism and predation.

**Results:**

We sequenced and assembled a high-quality genome of the dryinid wasp *Gonatopus flavifemur*, which at 636.5 Mb is larger than most hymenopterans. The expansion of transposable elements, especially DNA transposons, is a major contributor to the genome size enlargement. Our genome-wide screens reveal a number of positively selected genes and rapidly evolving proteins involved in energy production and motor activity, which may contribute to the predatory adaptation of dryinid wasp. We further show that three female-biased, reproductive-associated *yellow* genes, in response to the prey feeding behavior, are significantly elevated in adult females, which may facilitate the egg production. Venom is a powerful weapon for dryinid wasp during parasitism and predation. We therefore analyze the transcriptomes of venom glands and describe specific expansions in venom *Idgf*-like genes and neprilysin-like genes. Furthermore, we find the *LWS2-opsin* gene is exclusively expressed in male *G. flavifemur*, which may contribute to partner searching and mating.

**Conclusions:**

Our results provide new insights into the genome evolution, predatory adaptation, venom evolution, and sex-biased genes in *G. flavifemur*, and present genomic resources for future in-depth comparative analyses of hymenopterans that may benefit pest control.

**Supplementary Information:**

The online version contains supplementary material available at 10.1186/s12915-021-01081-6.

## Background

**Fig. 1:**
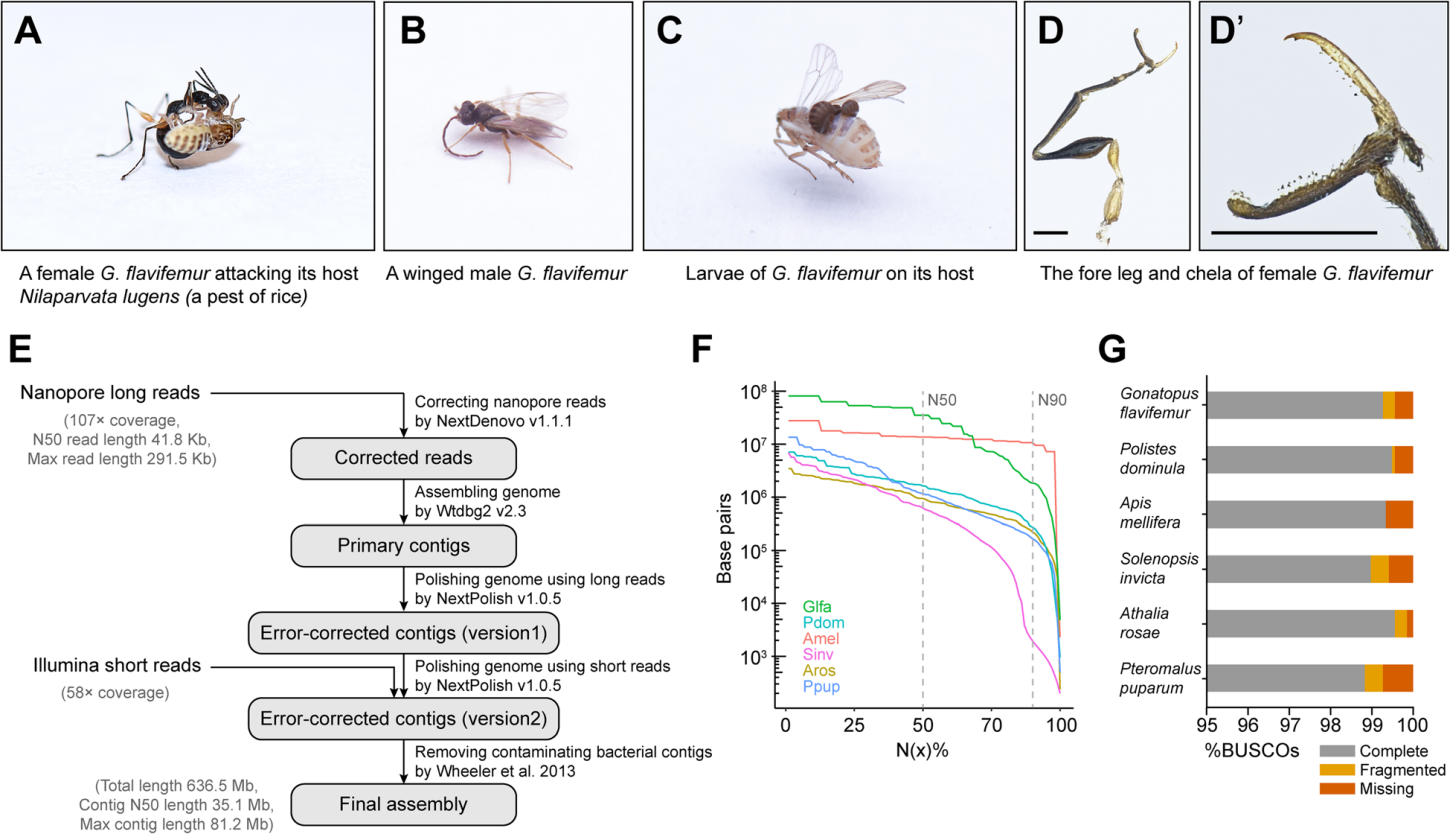
Assembly of the genome of *G. flavifemur.* **a** A female *G. flavifemur* attacking its host, the brown planthopper *Nilaparvata lugens*. **b** A winged male *G. flavifemur*. **c** Larvae of *G. flavifemur* on its host. **d, d’** The fore leg and chela of female *G. flavifemur*. Scale bars: 300 μm. **e** An overview of the genome assembly strategy. **f** Comparison of assembly contiguity among six hymenopterans. N(x) % graphs show contig or scaffold sizes (y-axis), in which x percent of the assembly consists of contigs/scaffolds of at least that size. **g** Comparison of the completeness of genome assemblies, as a percentage of 1367 insect genes from insecta_odb10

Hymenoptera are an extremely diverse insect order with a variety of life history traits, including phytophagy, parasitism, predation, pollination, and eusociality, providing an ideal model for studying the evolutionary origin and transition of some key traits [1, 2]. Dryinidae are a family within Chrysidoidea, which have several intriguing biological properties [3, 4]. These wasps are both parasitoids and predators of Auchenorrhyncha hosts (e.g., planthoppers) belonging to the order Hemiptera. Female wasps lay eggs on the hosts, and their young offspring develop outside the hosts (ectoparasitioid) (Fig. 1c). Also, female wasps catch and feed on the hosts. The protein-rich diets might be beneficial for egg production [4]. However, male wasps do not hunt or feed on hosts. The parasitoid wasps with both predatory and parasitoid behaviors are rare in Hymenoptera. Additionally, no other wasps with predatory behavior are found in Chrysidoidea [5]. Thus, the origin of predatory behavior in Dryinidae is likely an independent trait gaining event in the evolution of Hymenoptera. Moreover, Dryinidae are highly sexual dimorphic. The adult females are ant-like wasps and often wingless, whereas the adult males are winged [3] (Fig. 1a, b). Particularly, the fore legs of adult females evolved to be a pair of robust chelae, which are useful in prey capturing [3, 4] (Fig. 1d, d’). Therefore, dryinid wasp is also known as pincer wasp. The female adults have evolved a mimicry of ant-like body, which allows them to attack their hosts easily, as ants usually feed on the honeydew produced by Auchenorrhyncha insects [4]. Interestingly, these female-specific features seem to be related to their predatory behavior. However, very little is known about the genetic basis and evolutionary history of Dryinidae’s adaptation to their special parasitoid-predatory life.

Dryinidae provides a promising model to study the origin of predatory behavior and sexual dimorphism. *Gonatopus flavifemur* is a common parasitoid of the notorious rice pest, the brown planthopper *Nilaparvata lugens* [6–8]. Its unique predatory and parasitoid behaviors make this species very effective for the biological control of pests [9, 10]. Here, we report the genomic resources of *G. flavifemur*, representing the first genome sequence of the family Dryinidae. This 636.5 Mb genome assembly is much larger than most hymenopterans, due to the massive expansion of transposable element sequences. Analysis of the *G. flavifemur* genome highlights several positively selected genes and rapidly evolving proteins likely involved in major aspects of predatory adaptation. Gene expression changes in female adults after feeding on the preys are featured. Due to the importance of venom to the wasps, we also provide insights into the venom-associated genes, and describe specific expansions in venom *Idgf*-like genes and neprilysin-like genes. Finally, we identify many sex-biased genes which may be related to sexual dimorphism. In sum, our findings provide insights into the genome size evolution, parasitoid-predatory adaptation, venom evolution, and sexual dimorphism. In addition, this genome underpins further research of *G. flavifemur* and greatly facilitates future analyses of the trait evolution in Hymenoptera.

## Results

### Sequencing, assembly, and annotation

We generated Nanopore long reads (107X genome coverage) and Illumina short reads (58X genome coverage) from 50 male pupae for genome assembly (Fig. 1e; Additional file 1: Supplementary Table 1 – 4). The genome sizes of *G. flavifemur* estimated by flow cytometry and K-mer analysis were about 601.4 and 603.4 Mb, respectively (Additional file 1: Supplementary Table 5, Additional file 2: Supplementary Figure 1 – 2). After filtering out the bacterial contaminating contigs (1.7 Mb) (see Additional file 3 for details about the removal of bacterial contaminating contigs) [11–26], we obtained a 636.5 Mb high-contiguity genome assembly of *G. flavifemur*, with a contig N50 of 35.1 Mb. The maximum contig length reaches 81.2 Mb (Fig. 1e). We compared this genome assembly to other five high-quality genome assemblies in Hymenoptera, showing that our assembly has a higher contig N50 value (Fig. 1f). Although Hi-C or other technologies were not applied to improve our assembly to super-scaffold level, we found that the contig N50 value of our assembly is higher than the scaffold N50 values of two chromosome-level genomes in Hymenoptera *(Apis mellifera* and *Pteromalus puparum*) [13, 27]. Such very long contigs we obtained reflect the power of Nanopore long-read sequencing technology and related long-read-aware strategies in assembling a high-contiguity genome. Additionally, the appearance of very long contigs might be due to the larger genome size of *G. flavifemur*, which is 2.8 times the length of *A. mellifera* (255 Mb) and 1.9 times the length of *P. puparum* (338 Mb). Moreover, the haploid chromosome number of *Gonatopus* wasps is four [28]. Based on this information, the average chromosome length of *G. flavifemur* is 159.1 Mb, which is much larger than the maximum contig length of our assembly (81.2 Mb). Thus, the Nanopore long-read sequencing and assembly strategy, the large genome size, and low chromosome number explain the very long contigs obtained in our assembly.

Genome assessment using Benchmarking Universal Single-Copy Orthologs (BUSCO) indicated 99.3% of the insect gene set are present and complete (Fig. 1g). This BUSCO score is similar to other high-quality genomes. In addition, our analyses showed that 97.84% of Illumina whole-genome sequencing reads and 95.60% of RNA-seq reads could be properly mapped to the genome assembly respectively (Additional file 1: Supplementary Table 6). Thus, both BUSCO result and mapping quality indicated that our genome assembly is highly accurate and nearly complete. We annotated protein-coding genes by combining the evidence from homology alignments, de novo predictions and gene expressions. Totally, 23,100 protein-coding genes were identified in the genome. Comparing to a total number of 24,388 genes predicted in *Nasonia vitripennis*, our annotation is similar, but slightly larger than other hymenopterans. Moreover, we further identified several gene families that are important and popular in insect studies, including 66 cytochrome P450s, 17 glutathione S-transferases (GSTs), 35 ATP-binding cassette transporters (ABCs), 10 gustatory receptors (GRs), 20 ionotropic receptors (IRs), 43 olfactory receptors (ORs), 8 odorant binding proteins (OBPs), 8 sensory neuron membrane proteins (SNMPs), and 6 chemosensory proteins (CSPs) (Additional file 1: Supplementary Table 7). The gene numbers of detoxification-related gene families (P450, GST, ABC) in *G. flavifemur* are comparable to other hymenopteran insects we tested. However, in contrast to the detoxification genes, *G. flavifemur* has fewer chemosensory genes (OR, GR, OBP) than other hymenopteran insects, which may be explained by its relatively narrow host range (the main host is the brown planthopper, *N. lugens*) and unitary living environment (rice fields).

### Phylogenomics

**Fig. 2:**
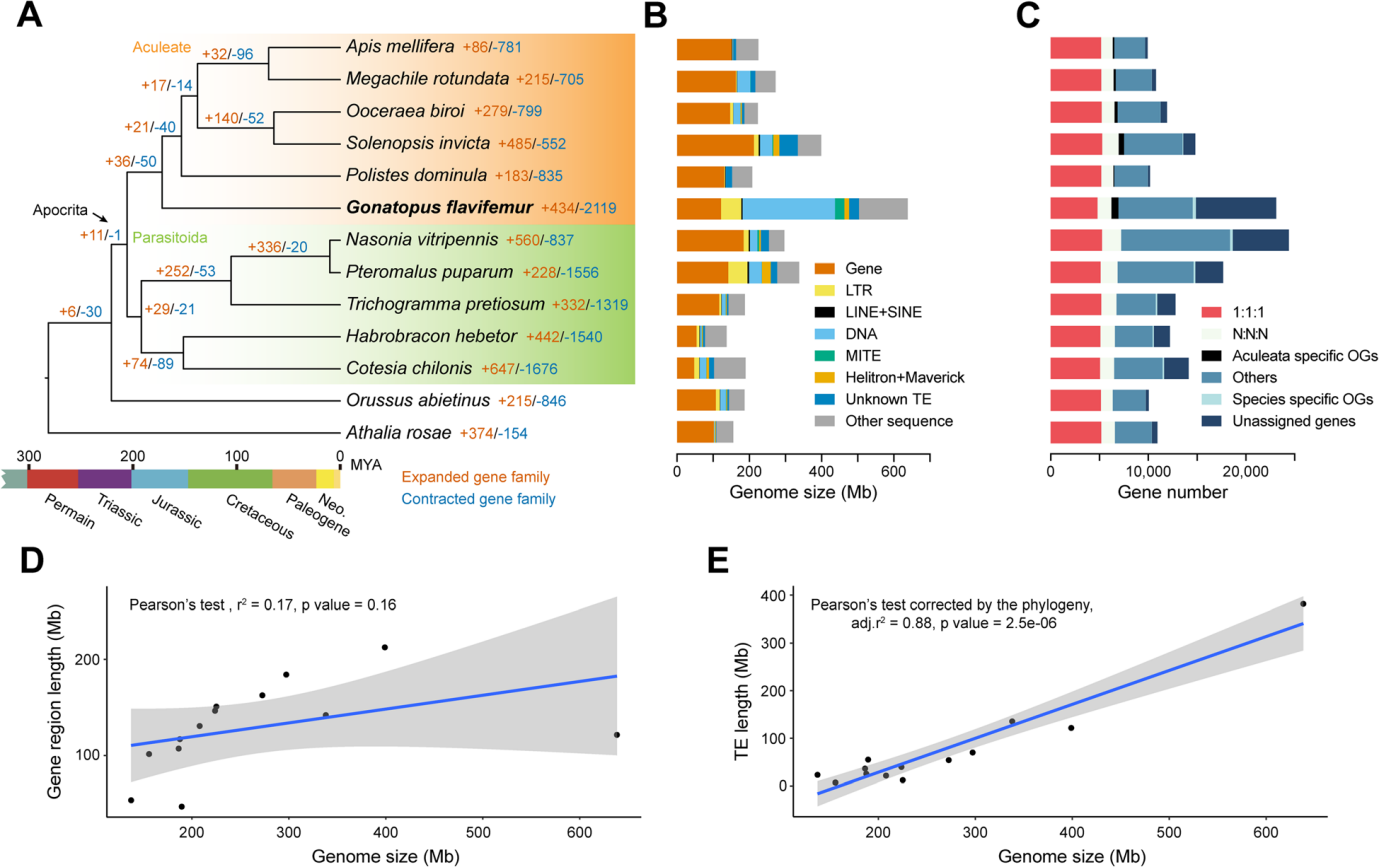
Phylogenetic and comparative genomic analysis of *G. flavifemur.* **a** The maximum likelihood phylogenetic tree built from 2992 concatenated single-copy orthologous groups from *G. flavifemur* and other 12 hymenopterans using IQ-TREE. The basal hymenopteran *A. rosae* was used as an outgroup. All nodes received 100% bootstrap support. The expansion numbers of gene families (orange) and contraction (blue) are shown on the branches. **b** Bar plots show total number of nucleotides occupied by genomic components. **c** Total gene counts of different types of orthologous groups in each genome. “1:1:1” indicates universal single-copy genes present in all species, absence and/or duplicated in, at most, one genome is included; “N:N:N” indicates other universal genes; “Aculeata specific OGs” indicates common unique genes in the six Aculeata species. “Species-specific OGs” represents species-specific genes with more than one copy in the genome. “Unsigned genes” indicates species-specific genes with only one copy in the genome. “Others” indicates the remaining genes. **d, e** The contribution of coding DNA sequence and TEs to genome size evolution across Hymenoptera. Lines correspond to linear regressions; shadows correspond to the 95% confidence intervals around the mean predictions

We chose 13 representative hymenopteran insects (including *G. flavifemur*) for phylogenomic analyses and the following comparative genomics analyses because of their high genome qualities, popularities, and evolutionary positions. Our phylogenomic analyses were based on 2992 single-copy genes, which were firstly identified by OrthoFinder from the genomes of 13 hymenopterans (one sawfly, one parasitic wood wasp, two braconid wasps, three chalcid wasps, one paper wasp, two ants, two bees, and one dryinid wasp). The amino acid sequences of the single-copy gene set were aligned and concatenated, followed by a phylogenetic tree construction. In this analysis, dryinid wasp *G. flavifemur* (from the superfamily Chrysidoidea) was placed as the sister group to all other Aculeate members (paper wasp, ants, and bees) after their common ancestor diverged from the infraorder Parasitoida (Fig. 2a, Additional file 2: Supplementary Figure 3a). In addition, we conducted coalescent-based analyses using ASTRAL by considering gene trees from the single-copy gene set individually, and obtained the same topology (Additional file 2: Supplementary Figure 3B). *G. flavifemur* diverged from other aculeates approximately 171.6 million years ago, during the Jurassic period (Fig. 2a). Our results indicate that the superfamily Chrysidoidea (including dryinid wasp) represents an early branch of the Aculeate, and this phylogenetic position is also supported by previous studies using transcriptome data and ultra-conserved elements [1, 2]. Filling the gap of the genomic sequences of this key phylogenetic position will greatly facilitate future comparative studies in Hymenoptera evolution.

### Genome size and transposable element

**Fig. 3:**
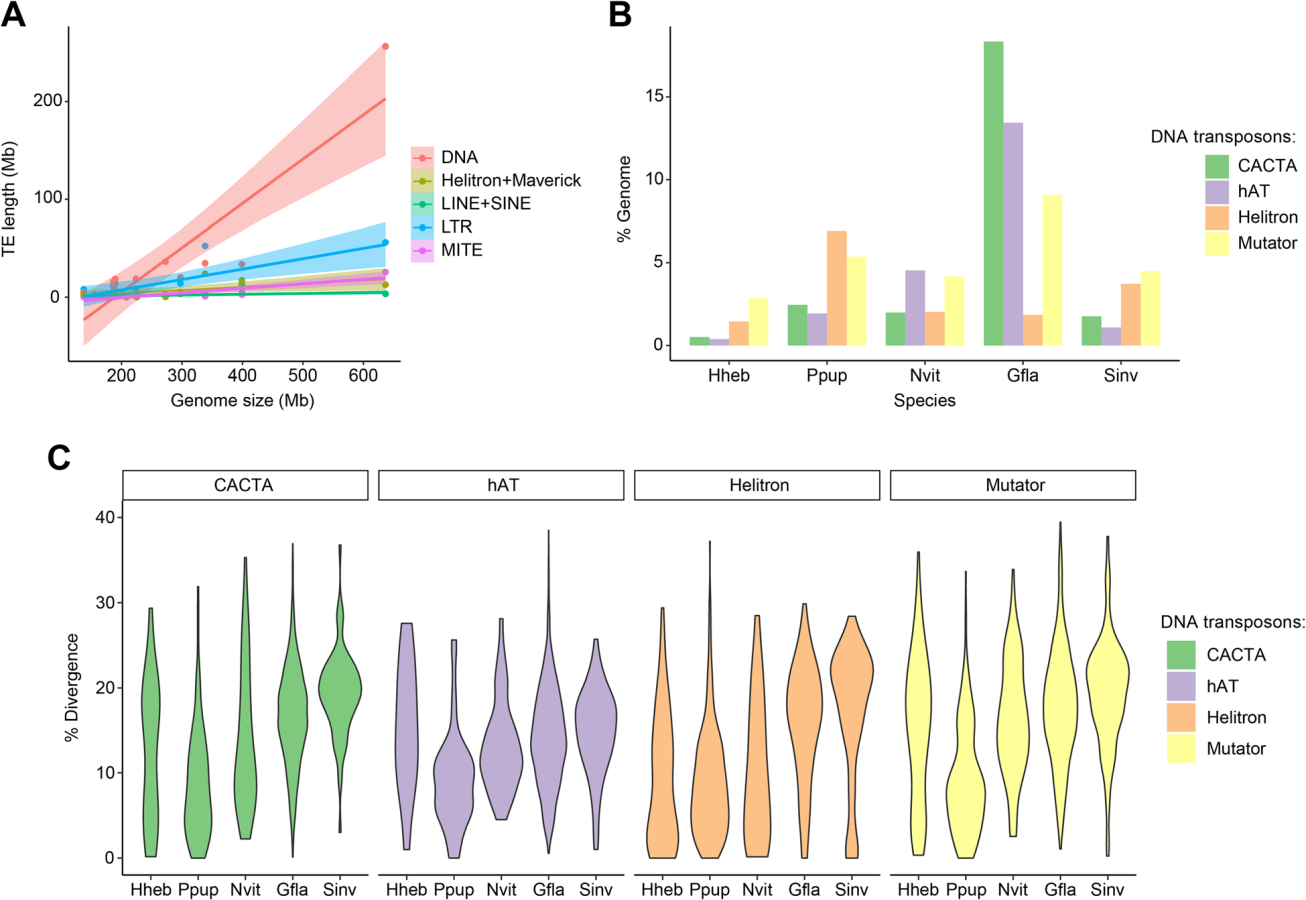
DNA transposons in the genome of *G. flavifemur*. **a** Correlation between total coverage of different TE classes and genome size across Hymenoptera. Lines correspond to linear regressions; shadows correspond to the 95% confidence intervals around the mean predictions. **b** The content of top four superfamilies of DNA transposons among five Hymenoptera species. **c** Violin plots showing each DNA transposon’s frequency distribution of sequence divergence level from the inferred ancestral consensus sequences. Gfla, *G. flavifemur*; Nvit, *N. vitripennis*; Ppup, *P. puparum.* Hheb, *H. hebetor*; Sinv, S. *invicta*

The hymenopteran genomes are moderate in size (80% are between 180 and 340 Mb) based on current sequencing projects [29]. However, there are a few exceptions [30], for example, the orchid bee *Euglossa dilemma* (3.3 Gb) [31] and the gall wasp *Belonocnema treatae* (1.5 Gb) (NCBI RefSeq assembly accession: GCF_010883055.1). The genome size of dryinid wasp *G. flavifemur* is 636.5 Mb, representing a relatively large genome in Hymenoptera. The expansion of repetitive sequences (e.g., transposable elements, TEs) is one of the most important factors to enlarge the genome size, and this phenomenon has been reported in many insects [30, 32–34]. Unsurprisingly, *G. flavifemur* genome contains massive repeat sequences, comprising approximately 60.7% of the whole genome. TEs account for around 59.9% (381.7 Mb) of the *G. flavifemur* genome, with DNA transposons being the most abundant TE group (40.3%; 256.3 Mb) (Fig. 2b). Based on the phylogeny of Hymenoptera, we noticed that TE content is strongly correlated with genome size (Fig. 2e; adj.r^2^ = 0.88, *p* = 2.5e−06, Pearson’s test corrected by the phylogeny), while gene region length shows weak correlation with genome size (Fig. 2d; adj.r^2^ = 0.17, *p* = 0.16, Pearson’s test). This result suggested that TE is a strong factor to drive genome size enlargement in the evolution of Hymenoptera. This pattern is supported by a previous study focused on aculeates [35]. The basal hymenopteran insect *Athalia rosae* (155.8 Mb) has a moderate genome size and a low TE content (4.6%), which may imply the ancestral state of hymenopteran genome. During the evolution of Hymenoptera, TE expansion likely happened independently after species divergence, resulting in genome size enlargement. In addition to the TE expansion of *G. flavifemur*, we found another obvious example, *P. puparum*, which also has abundant expanded TEs (about 40.1%) in its genome [13]. To figure out the contribution of each TE class to the genome size enlargement in Hymenoptera, we compared genome size differences with the content differences of each type of TE. The results showed that the expansion of DNA transposons to a large extent contributes to the genome size enlargement in Hymenoptera (Fig. 3a; adj.r^2^ = 0.77, *p* = 8.0e−05, Pearson’s test corrected by the phylogeny). We also detected moderate positive correlations between the content of long terminal repeat retrotransposons (LTRs) (adj.r^2^ = 0.35, *p* = 0.035, Pearson’s test corrected by the phylogeny), long interspersed nuclear elements (LINEs) and short interspersed nuclear elements (SINEs) (adj.r^2^ = 0.53, *p* = 0.0043, Pearson’s test corrected by the phylogeny), miniature inverted-repeat transposable elements (MITEs) (adj.r^2^ = 0.40, *p* = 0.021, Pearson’s test corrected by the phylogeny), and the genome size. This result suggested that the expansions of other TEs (LTRs, LINEs, SINEs, and MITEs) also contribute to genome size evolution in Hymenoptera.

In *G. flavifemur*, 40.3% of the genome consists of DNA transposons, whereas the DNA transposons contents in other hymenopteran insects are much lower (0.2 to 13%) (Additional file 1: Supplementary Table 8). The DNA transposon expansion might be largely responsible for the genome size increase in *G. flavifemur*. To further investigate the main contributors of TE expansion, we identified four most abundant superfamilies of DNA transposons in the *G. flavifemur* genome: CACTA (18.3%, Superfamily code: DTC), hAT (13.4%, Superfamily code: DTA), Mutator (9.1%, Superfamily code: DTM), and Helitron (1.9%, Superfamily code: DHH) (Fig. 3b; Additional file 1: Supplementary Table 9). Although *P. puparum* has the highest Helitron content among five hymenopterans, the contents of CACTA, hAT, and Mutator superfamilies in the *G. flavifemur* genome were much higher than that in other genomes. We also found the divergence levels of these TE superfamilies are generally lower than 20% among five hymenopteran insects, suggesting that they may be recently active in the genomes (Fig. 3c). Compared with the other four genomes, *G. flavifemur* lacks shared patterns in terms of TE divergences. In addition, the closest relative of *G. flavifemur* that has been sequenced is *Goniozus legneri* (family Bethylidae, superfamily Chrysidoidea), with a small genome (140.1 Mb) and low TE content (7.8%; Additional file 1: Supplementary Table 8) [36]. However, the genome of *G. legneri* was poorly assembled with a scaffold N50 of 167.3 kb, so this genome was not included in other analyses of the paper [36]. Thus, we concluded that the expansion of DNA transposons, mainly from CACTA, hAT, and Mutator superfamilies, occurred in *G. flavifemur* has predominantly enlarged its genome, after *G. flavifemur* diverged from *G. legneri* about 162 million years ago [1].

### Gene content comparison

We identified 12,696 orthogroups (OGs) using OrthoFinder among *G. flavifemur* and the 12 other hymenopteran insects used in our analyses. Of these, 4608 OGs (5281 *G. flavifemur* genes) are present in all hymenopteran insects analyzed in this study. The *G. flavifemur* genome contains 727 Aculeate-specific genes, which is more than other aculeates. GO enrichment analysis revealed that these Aculeate-specific genes are enriched in DNA integration, DNA recombination, cellular aromatic compound metabolic process, and nitrogen compound metabolic process (*p* < 0.05, false discovery rate adjusted, FDR-adjusted; Additional file 1: Supplementary Table 10). A total of 8453 genes from the *G. flavifemur* genome were species-specific and among which, 2516 showed BLAST hits against the NCBI nr database, suggesting that they may have homologs outside the phylogenetic context of our comparative genomics study. The rest of 5937 genes were inferred as species-specific and/or orphan in *G. flavifemur*. These 5937 genes were enriched in GO terms related to serine hydrolase activity and phosphorus-nitrogen bonds hydrolase activity (*p* < 0.05, FDR-adjusted; Additional file 1: Supplementary Table 11).

### Gene selection and gene family evolution in *G. flavifemur*

Unlike most of the parasitoid wasps in the infraorder Parasitoida (e.g., braconid wasps and chalcid wasps), the dryinid wasps (only female wasps) are usually both parasitoids and predators on their hosts [8]. As parasitoids, females lay eggs on the live hosts, and their larvae hatch and feed on the host. As predators, females catch the hosts and feed on the hemolymph and tissues directly [8]. The hosts of dryinid wasps are from the suborder Auchenorrhyncha (order Hemiptera), most of which are planthoppers and leafhoppers. They are among the extremely agile insects, and adept at jumping and flying. To increase the success rate of parasitism and predation, female wasps are morphologically distinct from the males, including elongated and very mobile prothorax, a pair of chelae-like fore legs, and an ant-like, wingless body [4]. Predation behavior is universal in aculeate insects, such as ants, vespids, spider wasps, and dryinid wasps [5]. However, the predation mode of the dryinid wasps is different from most aculeates. Female dryinid wasp hunts and feeds directly on hosts (i.e., adult predacious feeding), while many aculeates paralyze their prey and transport it to their nests for larval feeding (i.e., provisioning predators) [5]. As a member of superfamily Chrysidoidea, dryinid wasp (family Dryinidae) represents the only case of predation, suggesting an independent origin of predatory behavior in Dryinidae. To gain insight into the genomic basis of the *G. flavifemur*-specific evolutionary traits, including the origin of predation, we performed genome-wide selection analysis and gene family evolutionary analysis to screen for gene changes that occurred in the *G. flavifemur* genome.

First, 2992 single-copy genes were used for screening the signatures of positive selection on the terminal branch of *G. flavifemur* in the phylogeny in Fig. 2a. Here, two tests (aBSREL model in HyPhy and branch-site model in PAML) were used (*p* < 0.05, FDR-adjusted) to define the significance of each candidate gene. Only those genes showing positive selection signals in both methods were used for the following study. In total, our strict screening criteria identified 183 genes that have evidence of positive selection in the *G. flavifemur* genome (Additional file 1: Supplementary Table 12). We did not observe any significantly enriched GO terms for these positively selected genes, suggesting that the functions of these genes might be diverse (*p* > 0.05, FDR-adjusted). We found some positively selected genes are related to mitochondrial functions, including *NDUFB3*, *NDUFA9*, *mtTFB1*, *Myg1*, and *PTCD3* [37–40] (Additional file 1: Supplementary Table 12). Among them, two genes (*NDUFB3* and *NDUFA9*) are in the mitochondrial electron transport chain (Complex I), which transfers electrons from NADH to ubiquinone [37]. Given the critical role of mitochondria in cellular respiration and energy production, these mitochondrial-related positively selected genes might function in providing energy for *G. flavifemur*’s hunting. In addition, a number of positively selected genes are involved in actin cytoskeleton, muscle contraction, and motor activity, including *TLN1* [41], *TBCE* [42], *Scgβ* [43], *SPG11* [44], *ALS2* [45], *TWF1* [46, 47], and *SIM* [48] (Additional file 1: Supplementary Table 12). These may also contribute to the predation behavior of *G. flavifemur*. Interestingly, our positively selected gene set includes a Piwi-like *AGO3* gene, which belongs to the piRNA pathway. This AGO3 protein interacts with piRNA and plays a central role during meiosis by repressing TEs and preventing their mobilization, which is essential for germline integrity [49–52]. Our results above have indicated that TEs massively expanded in the *G. flavifemur* genome and some TEs may still be active. Thus, we hypothesize that this selected *AGO3* of *G. flavifemur* might have functions on repressing TEs in the germline.

Additionally, we performed a rank-based branch length comparison method to study the protein evolutionary rates of the *G. flavifemur* branch. Amino acid substitutions provide strong evidence to fast or slow evolution when the divergence time is very long and the synonymous substitutions may be saturated. This method was used to identify rapidly evolving proteins in the *Trichogramma* wasp genome [14]. In this analysis, we reconstructed the phylogenetic tree of each of the 2992 single-copy proteins, and then for each protein, we extracted the total branch length and the terminal branch length of *G. flavifemur*. The total branch length represents the general protein evolution pattern of each protein, while the terminal branch length of *G. flavifemur* represents the protein evolution rate after *G. flavifemur* diverged from other species. Here, we first assigned ranks to proteins (from 1 to 2992) based on their total branch lengths (i.e., total branch length rank). Then, we assigned ranks to proteins (from 1 to 2992) based on their branch lengths of *G. flavifemur* branches (i.e., *G. flavifemur* branch length rank). We next binned proteins into groups of 300 based on their total branch length rank, which could avoid overrepresentation among any protein categories based on general evolutionary rates. In each bin, we selected the proteins with the top 10% largest discrepancy in rank between the *G. flavifemur* branch length rank and the total branch length rank, as rapidly evolving proteins (i.e., we chose the proteins with the top 10% largest values of the *G. flavifemur* branch length rank minus the total branch length rank) (Additional file 2: Supplementary Figure 4). We found that the rapidly evolving protein set is largely divergent from the positively selected gene set, with only 18 genes overlapped. In these rapidly evolving proteins, we found additional proteins involved in respiratory electron transport chain, which are ETFRF1 and NDUFB7 [37, 53]. Furthermore, additional proteins which may contribute to motor activities were found, including profilin [54], cGMP-dependent protein kinase (cGK) [55], and two-pore potassium channel protein sup-9 [56]. Four proteins from the 20S core proteasome complex were identified as rapidly evolving proteins, suggesting a potentially important role of proteasomes in *G. flavifemur*. One of these four genes is also in the positively selected gene set. In addition, three ABC transporter proteins (related to detoxification), one visual system homeobox 2 protein (related to visual perception), and one cryptochrome-1 protein (related to circadian rhythm) were identified. Together, our searches for positively selected genes and rapidly evolving proteins revealed several genes or proteins involved in energy production and motor activity, which suggests that altered genes or proteins may contribute to the adaptation of predatory behavior in *G. flavifemur*.

We next investigated the gene family expansions and contractions in the *G. flavifemur* genome. A total of 434 gene families were expanded in *G. flavifemur* comparing to the common ancestor of the Aculeate, including chitinase-like proteins (glycosyl hydrolase family 18), neprilysins (zinc-dependent metalloproteases), trypsins, venom carboxylesterases, esterases, G-protein-coupled receptors, and several transcription factors (Fig. 2a). The significantly enriched GO terms included digestion and regulation of skeletal muscle adaptation, which may also associate with the predation behavior (*p* < 0.05, FDR-adjusted; Additional file 1: Supplementary Table 13). The *GTF2IRD* (general transcription factor II-I repeat domain-containing protein) genes were expanded in *G. flavifemur*, and they may contribute to slow-twitch fiber type specificity during myogenesis and regenerating muscles [57]. Interestingly, we found a number of expanded genes from chitinase-like gene family (glycosyl hydrolase family 18) and neprilysin family were much more highly expressed in the venom gland of *G. flavifemur* than in carcass (i.e., adult female tissues minus the venom gland), adult male, pupa, and larva, suggesting that they might be venom genes and play important roles in parasitoid-host interactions. See below for the detailed analyses about the chitinase/chitinase-like gene family and the neprilysin family.

### Venom gland-associated genes of *G. flavifemur*

Among most of the hymenopteran insects, venom plays essential roles in their life. For example, parasitoid wasps use venom to manipulate the metabolism and immunity, and gene expression of the host to establish a suitable environment for wasp larvae [58–64], while some predatory hymenopterans (e.g., vespid wasps and ants) use venom for prey capture and defense [63, 65, 66]. The venom components of several parasitoid wasps and predatory hymenopterans have so far been reported [61, 65–70]. However, little is known about the venom components and their functions of *G. flavifemur*, which acts as both parasitoid and predator on its host. We hypothesize that the venom of *G. flavifemur* might have similar functions with the venoms of both parasitoid wasps and predatory wasps, and it is expected to play a key role in both altering host’s metabolism and immunity (like many parasitoid wasps’ venom), as well as paralyzing the host temporarily (like many predatory wasps’ venom).

In order to gain insights into the putative venom gland-associated genes (VGGs) of *G. flavifemur*, we analyzed the RNA-seq datasets derived from venom gland and carcass (i.e., adult female tissues minus the venom gland) respectively. The venom gland is a highly specialized organ, which produces the venom components. We found that there is only a small set of genes highly expressed in the venom gland. For example, only 157 and 474 genes account for 80% and 90% of expression in the venom gland of *G. flavifemur*; however, 979 and 2297 genes account for 80% and 90% of expression in adult female (Additional file 2: Supplementary Figure 5A). This pattern could also be found in the venom gland of many other parasitoid wasps [61]. Based on the transcriptome data, we used three major criteria to identify VGGs in *G. flavifemur*, (1) VGGs must be among the top500 expressed genes (ranked by the median FPKM values) in the venom gland transcriptome; (2) VGGs must be significantly highly expressed in the venom gland relative to the carcass (*q* < 0.05); (3) VGGs must remain low expression levels in the carcass (median FPKM < 50). In total, 154 VGGs were identified in *G. flavifemur* (Additional file 1: Supplementary Table 14). These 154 VGGs have significantly higher expression levels in the venom gland and low expression levels in other developmental stages (*p* < 2.2e−16, Wilcoxon rank-sum test; Additional file 2: Supplementary Figure 5B). Among these 154 VGGs, 32 genes have predicted signal peptides (Additional file 1: Supplementary Table 14), which indicates the presence of secretory signals and is considered as one of the characteristics of venom proteins in previous studies [68, 69]. However, signal peptide may not be necessary for a venom protein, since evidence has shown that some RNA-seq supported proteins (encoded by venom gland highly expressed genes) without signal peptides can also be detected in venom proteomes [61, 68]. Moreover, venom-related extracellular vesicles (i.e., venosome) were found in some wasps, which may directly transport venom proteins to their targets in hosts, even if they do not contain signal peptides [71]. The VGGs contain a broad range of functional components, such as proteases and peptidases (46 genes, 29.9%), protease inhibitor (1 gene, 0.7%), lipases (10 genes, 6.5%), chitinase-like genes (29 genes, 18.8%), and oxidoreductases (3 genes, 1.9%) (Additional file 2: Supplementary Figure 5C). Some of them are known venom proteins of other parasitoid wasps, including serine proteases, serpins (protease inhibitors), phospholipases, and major royal jelly proteins [67, 68].

Notably, a number of chitinase-like genes (29 genes, 8 genes with predicted signal peptides) were found to be specifically highly expressed in the venom gland of *G. flavifemur* (the mean FPKM in the venom gland is 4228.5, while the mean FPKM in the carcass is 5.4). Such a large number of venom-expressed chitinase-like genes was not reported in any other wasps, and might play an important role during the parasitism and hunting of *G. flavifemur*. It could occur due to the gene duplications after a single venom gene recruitment, or many independent venom gene recruitment events after gene duplications, or a complex evolutionary history that includes both cases above. Additionally, the venom gene recruitment of chitinase-like genes might be due to the lateral gene transfer (LGT), and it has been reported in some parasitoids in Chalcidoidea [72]. However, we did not find any evidence to support the hypothesis that some of these highly expressed chitinase-like genes in the venom gland were laterally transferred from bacteria or fungi.

Because of the abundant chitinase-like genes and clotting-related proteases (8 genes) in the venom gland, the GO enrichment analysis showed that the VGGs are enriched in carbohydrate metabolic process, imaginal disc development, coagulation, wound healing, proteolysis, cuticle development (*p* < 0.05, FDR-adjusted; Additional file 1: Supplementary Table 15 and Additional file 2: Supplementary Figure 5D). It seems that the venom of *G. flavifemur* might regulate the host’s cuticle development and prevent the host from dying due to excessive hemolymph loss. Parasitized hosts will be alive until the mature wasp larvae leave the hosts for cocooning and pupation [4]. Therefore, it is reasonable to hypothesize that *G. flavifemur* venom might be involved in the wound healing process to ensure the host is alive.

There are total 26 neprilysin-like genes (M13 peptidases) in the VGGs, and 7 of them contain predicted signal peptides (Additional file 1: Supplementary Table 14). Neprilysin is a zinc-dependent metalloprotease with a broad range of physiological targets, including natriuretic, vasodilatory, and neuro peptides [73]. Venom neprilysins have been reported in many venomous animals, such as jellyfishes, snakes, spiders, and solitary hunting wasps *Eumenes decorates* and *Ampulex compressa* [70, 74–78]. The functions of venom neprilysins are related to neurotoxicity which can paralyze the prey immediately [79, 80]. Such high abundant venom neprilysin-like genes may imply that paralysis might be one of the major functions of the venom of *G. flavifemur*. Moreover, we found a hemolymph lipopolysaccharide-binding protein gene in the VGG set, which may play a role in bacterial clearance and protect parasitoid larvae from the influence of bacteria [81]. The hemipteran hosts of *G. flavifemur* usually carry some intracellular symbionts [82]. It would be interesting to study if this venom lipopolysaccharide-binding protein could target the host’s bacterial symbionts.

### Expansion and venom expression of chitinase-like genes in *G. flavifemur*

**Fig. 4:**
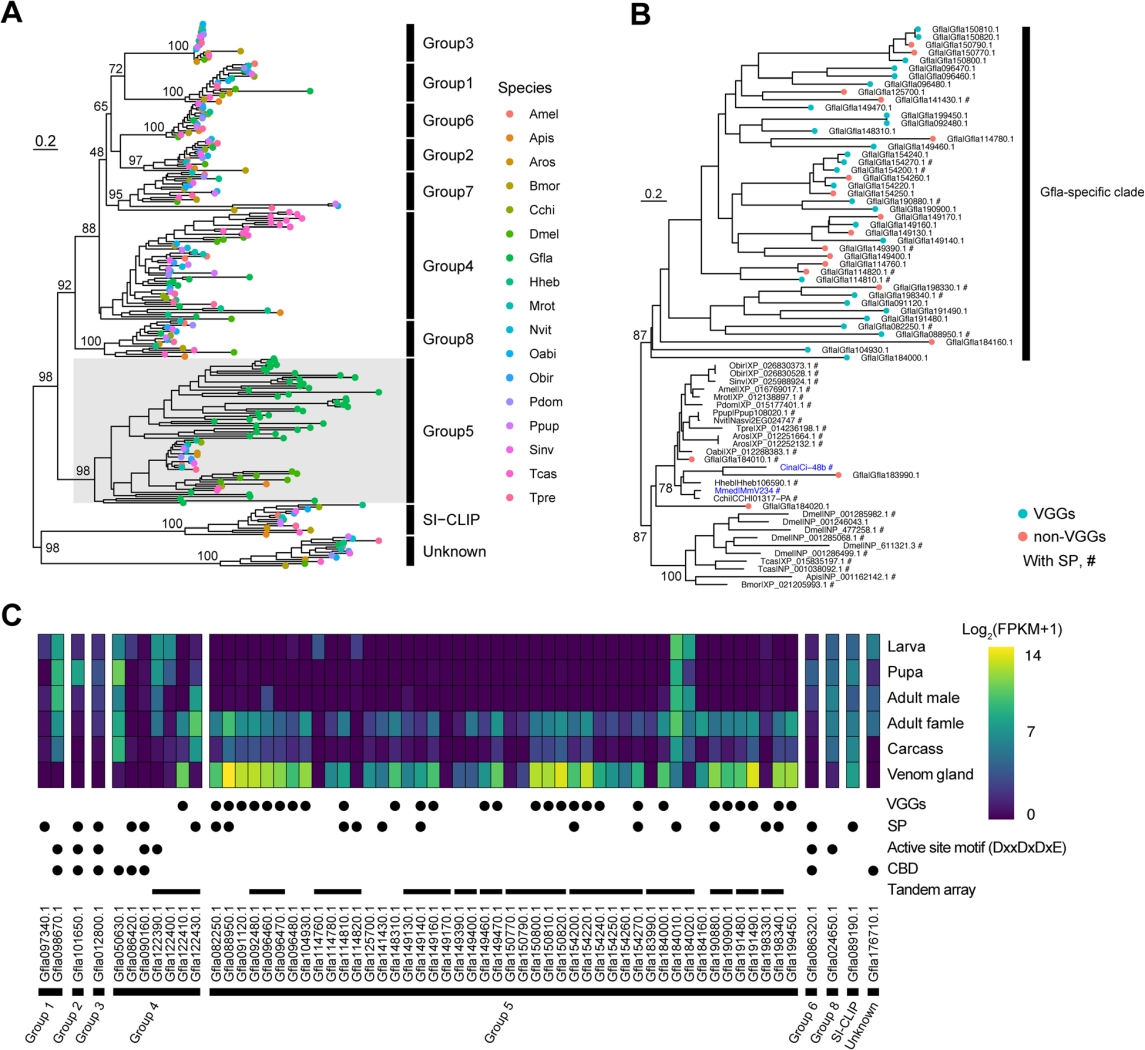
Chitinase-like genes in the *G. flavifemur* genome. **a** The maximum likelihood phylogenetic tree of chitinase-like proteins in *G. flavifemur* and other 16 insect species. The tree was built using IQ-TREE and branch support was assessed by 1000 ultrafast bootstrap replicates. Different groups of chitinase-like genes are indicated according to the descriptions in Arakane and Muthukrishnan [88]. **b** The maximum likelihood phylogenetic tree of imaginal disc growth factors (IDGFs) built by IQ-TREE. The branch support was assessed by 1000 ultrafast bootstrap replicates. The *G. flavifemur*-specific clade of IDGFs was shown. Two additional venom IDGFs from two parasitoid wasps *Microplitis mediator* and *Chelonus inanitus* were labeled in blue. **c** The expression pattern of chitinase-like genes in *G. flavifemur* across development and different tissues. Signal peptide (SP), VGGs (venom gland-associated genes), active site motif (DxxDxDxE), chitin biding domain (CBD) and tandem array were indicated

To gain more insights into the expansion of the chitinase-like gene family (glycoside hydrolase 18 family, GH18) in *G. flavifemur*, we searched GH18 domain-containing proteins in 17 insect genomes, which includes 13 hymenopteran genomes (including *G. flavifemur*), 1 lepidoptera genome (*Bombyx mori*), 1 coleopteran genome (*Tribolium castaneum*), 1 diptera genome (*Drosophila melanogaster*), and 1 hemipteran genome (*Acyrthosiphon pisum*). The four non-hymenopteran genomes we analyzed here allow us to determine the ancestral state of chitinase-like gene family of Hymenoptera. In total, 260 GH18 domain-containing proteins were found and 61 of them were in the *G. flavifemur* genome, representing the most abundant one when compared to the other 16 genomes. Maximum likelihood phylogenetic analysis indicated that the chitinase-like genes in group 4 (7 genes) and group 5 (47 genes) were expanded in the *G. flavifemur* genome, whereas the gene counts in other chitinase groups among 17 insect genomes were highly conserved (Fig. 4a, b).

Group 5 chitinase-like proteins are also annotated as imaginal disc growth factors (IDGFs), which are a small family of chitinase-related secretory proteins found in many insects [83–87]. They lack chitinase activity due to an amino acid substitution of a key glutamate residue (E) in a conserved active site motif [88]. However, a few examples in *T. castaneum* and parasitoid wasp *Microplitis mediator* and *Cotesia chilonis* show that although IDGFs retain the glutamate residue in the active site motif, a D to A substitution in the same motif (from DxxDxDxE to DxxDxAxE) also results in the loss of function of IDGFs (Additional file 2: Supplementary Figure 6) [88]. In *G. flavifemur*, we identified 47 *Idgf* genes. This number is much higher than any other insects we surveyed, including *G. legneri*, the closest relative of *G. flavifemur* (Additional file 1: Supplementary Table 16). Phylogenetic analysis showed that *Idgf* genes were largely expanded in the *G. flavifemur* genome, and 44 of total 47 *Idgf* genes were clustered together (Fig. 4b). We found a well-supported (87% of rapid bootstraps) insect conserved *Idgf* clade, which contains three *G. flavifemur Idgf* genes. In total, 44 *Idgf* paralogs in the *G. flavifemur* genome clustered together to form a *G. flavifemur*-specific clade instead of clustered with the *Idgf* ortholog in insect conserved *Idgf* clade, suggesting a rapidly evolutionary history of these paralogs (Fig. 4b). Gene distribution analysis revealed many *Idgf* gene tandem arrays (with 2 or more *Idgf* genes) in the *G. flavifemur* genome, indicating the tandem duplications of *Idgf* genes. The largest tandem array contains 6 *Idgf* genes (Fig. 4c). Gene expression patterns showed that the three *G. flavifemur Idgf* genes in insect conserved *Idgf* clade expressed widely across development, while many *Idgf* genes in *G. flavifemur*-specific clade display strong venom-gland biased expression patterns and lowly express in adult male, pupa, and larva (Fig. 4c). Due to the low pairwise identity, with only 27.22% on average, we concluded that the cross-mapping events among these genes are neglectable during RNA-seq analysis. Interestingly, we observed an *Idgf* tandem array, which includes four genes. Three of them are insect conserved *Idgfs* (no venom expression) while the last one is *G. flavifemur*-specific. Moreover, this *G. flavifemur*-specific *Idgf* displayed a strong venom-gland biased expression pattern. Among 47 *Idgf* genes, 28 were assigned as VGGs in our analysis (Fig. 4b, c). Gene family expansion and venom recruitment of *Idgf* genes in *G. flavifemur* implied that these genes may have important functions as venom components, although only 12 of 28 venom gland-associated *Idgf* genes have predicted signal peptides. Venom IDGF proteins are unusual in hymenopterans and have been reported in only two additional parasitoid wasps, *M. mediator* and *Chelonus inanitus* [89, 90]. These venom IDGF proteins (MmV234 and Ci-48b) were clustered with other hymenopteran IDGFs in the insect conserved *Idgf* clade (Fig. 4b).

An additional gene from the group 4 chitinase subfamily was also highly expressed in the venom gland and very lowly expressed across development (Fig. 4c). This gene is located in a tandem array of group 4 chitinase genes, showing a tandem duplication event. Together, our observations imply that the expanded *Idgf* and group 4 chitinase genes with venom-gland high expressions might have important functions in *G. flavifemur*-host interaction. Further studies are needed to investigate the function of these venom chitinase-like genes.

### Expansion and convergent venom recruitment of neprilysin-like genes in *G. flavifemur*

**Fig. 5:**
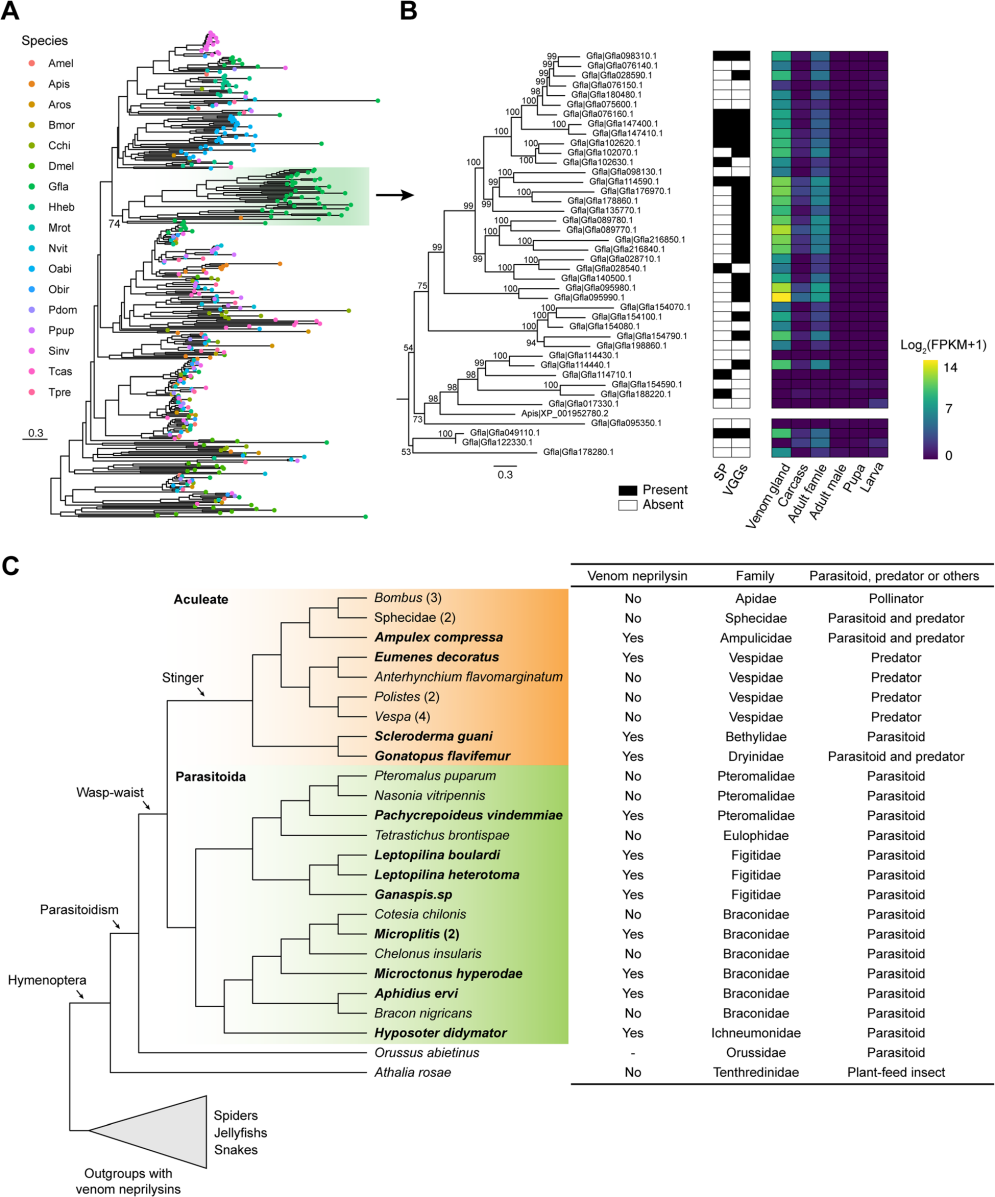
Neprilysin-like genes in the *G. flavifemur* genome. **a** The maximum likelihood phylogenetic tree of neprilysin-like proteins in *G. flavifemur* and other 16 insect species. The tree was built using IQ-TREE and branch support was assessed by 1000 ultrafast bootstrap replicates. **b** The phylogenetic tree of neprilysin-like genes in the *G. flavifemur*-specific clade and their expression pattern in venom gland, adult female tissues minus the venom gland (i.e., carcass), adult female, adult male, pupa, and larva. SP, signal peptide; VGGs, venom gland-associated genes. **c** Venom recruitment of neprilysin-like genes in Hymenoptera. Totally, the venom components of 31 hymenopterans were exanimated (15 in Parasitoida and 16 in Aculeate). The ectoparasitoid wood wasp *O. abietinus* and the non-venom basal hymenopteran *A. rosae* were also included. The phylogeny of these hymenopterans was obtained from previous studies [2, 91]. Others animals, such as snakes, spiders, which carry venom neprilysin-like genes were shown

In the *G. flavifemur* genome, we identified 66 neprilysin-like genes (containing Peptidase_M13 domain), which is much more abundant than any other insects in this study, ranging from 5 to 42 (Additional file 1: Supplementary Table 17). Phylogenetic analysis further confirmed that neprilysin-like genes were largely expanded in the *G. flavifemur* genome, and 41 neprilysin-like genes were clustered together to form a *G. flavifemur*-specific clade (Fig. 5a, b). Notably, this *G. flavifemur*-specific clade includes 23 of total 26 venom gland-associated neprilysin-like genes defined by the venom gland transcriptomes (Fig. 5b). Neprilysin is a zinc-dependent metalloprotease and is predicted to metabolize regulatory peptides in prey’s nervous system and paralyze the prey as a venom protein [79, 80]. The expansion and venom high expression of these neprilysin-like genes suggest that the *G. flavifemur* venom might have a powerful paralyzing effect on the host. Additionally, we identified a large expansion of the *Orussus abietinus* neprilysin-like genes (Fig. 5a). *O. abietinus* is an ectoparasitoid of xylobiontic larvae of beetles or wood wasps [35]. In phylogeny, *O. abietinus* represents the closest relative of Apocrita, which may have a similar lifestyle with the parasitoid ancestor [2]. It is worth investigating whether the expanded neprilysin-like genes are also highly expressed in the venom of *O. abietinus*.

Venom neprilysins have been reported in many venomous animals, such as jellyfishes, snakes, and centipedes and spiders, suggesting many convergent venom recruitments [74, 76, 78, 80]. We next asked whether the neprilysin-like genes are present in the venoms of other species in Hymenoptera by convergent recruitments. To this end, we examined the reported venom components of 30 additional hymenopterans (15 in Parasitoida and 15 in Aculeate) to search venom neprilysin-like genes (Fig. 5c). In total, including *G. flavifemur*, we found venom neprilysin-like genes in 13 wasps (9 in Parasitoida and 4 in Aculeate). In Parasitoida, venom neprilysin-like genes were found in a chalcid wasp *Pachycrepoideus vindemmiae*, and 3 figitid wasps *Leptopilina boulardi*, *L. heterotoma*, and *Ganaspis* sp., 4 braconid wasps *M. mediator*, *M. demolitor*, *Microctonus hyperodae*, *Aphidius ervi*, and an ichneumonid wasp *Hyposoter didymator*. In Aculeate, we found additional venom neprilysin-like genes in a bethylid wasp *Scleroderma guani*, a solitary hunting wasp *Eumenes decorates* and an emerald cockroach wasp *Ampulex compressa*. We then summarized the presence or absence of the venom neprilysin-like genes in total 31 hymenopterans and mapped the information to their phylogeny. Our results show that the recruitments of venom neprilysin-like genes are mainly scattered throughout the Hymenoptera phylogeny. This indicates that most recruitment events of venom neprilysin-like gene occurred independently during the radiation of Hymenoptera (i.e., convergent venom recruitments).

### Gene expression changes in female adults after feeding on the preys

**Fig. 6:**
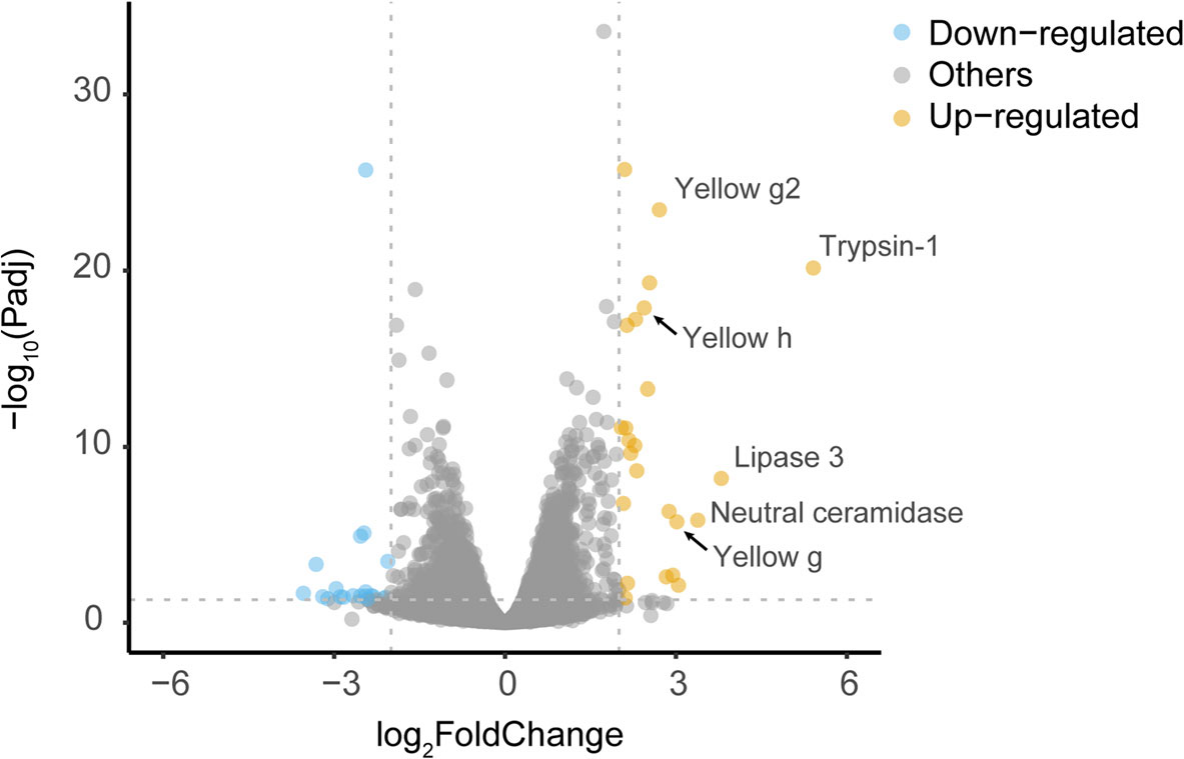
Volcano plot showing differential gene expression between prey-feeding females and sucrose-feeding females of *G. flavifemur.* In total, 24 significantly upregulated genes (shown in yellow) and 20 significantly downregulated genes (shown in blue) were found (fold change > 4 and *q* < 0.05). Importantly, three *yellow* genes (*yellow-g*, *yellow-g2*, and *yellow-h*) significantly increased their expressions (5.44–8.09-fold higher expression) after feeding on the preys

Some female insects, such as blood-feeding mosquitoes, need to feed on proteins to trigger egg development [92]. A previous study reported that female dryinid wasps often consumed the first host captured in the day [5]. We then hypothesized that the hunting and prey feeding behaviors of *G. flavifemur* female adults may be beneficial to reproduction. To test our hypothesis, we analyzed the transcriptome data derived from the prey-feeding females (protein-rich diet) and sucrose-feeding females to characterize differential expressed genes between these two treatments. In total, 24 genes were significantly upregulated in the prey-feeding females when compared to the sucrose-feeding females (fold change > 4 and *q* < 0.05; Additional file 1: Supplementary Table 18). We observed that 3 *yellow* genes were significantly activated (5.44–8.09-fold higher expression) after feeding on the preys (Fig. 6). Compared with other insect *yellow* genes, phylogenetic analysis suggested that these 3 activated *yellow* genes belong to subfamily *yellow-g*, *yellow-g2*, and *yellow-h*, respectively (Additional file 1: Supplementary Table 19). *Yellow* genes are common within arthropods and are homologous with major royal jelly protein encoding genes [93, 94]. In some well-studied insects, such as fruit fly, mosquito, and silkworm, some members of the *yellow* gene family are associated with reproductive maturation [95–97]. In *G. flavifemur*, analyses of transcriptome data of male and female adults showed that the 3 upregulated *yellow* genes in the prey-feeding females are extremely female-biased genes, with 266.98–1656.33-fold higher expression in the female adults than in the male adults (*q* < 0.05; Additional file 1: Supplementary Table 19). Therefore, our finding indicates that 3 female-biased *yellow* genes are significantly upregulated in females after feeding on the preys, and these genes may be involved in the reproduction of *G. flavifemur*. Other significantly upregulated genes in the prey-feeding females including trypsin-1, lipase 3, neutral ceramidase, *takeout*, and fatty acyl-CoA reductase (Fig. 6 and Additional file 1: Supplementary Table 18). We also identified 20 significantly downregulated genes in the prey-feeding females when compared to the sucrose-feeding females, which included maltase2, growth arrest-specific protein 8, liver carboxylesterase, and small heat shock protein C4 (fold change > 4 and *q* < 0.05; Additional file 1: Supplementary Table 20).

### Sex-biased genes and male-biased opsin genes in *G. flavifemur*

Due to the obvious sexual dimorphism of *G. flavifemur*, we also investigated the sex-biased genes in this wasp by comparing the RNA-seq data of adult males and females. The 461 extremely female-biased genes (fold change > 16 and *q* < 0.05) were enriched in nucleosome assembly, meiotic cell cycle process (*p* < 0.05, FDR-adjusted; Additional file 1: Supplementary Table 21). However, the 362 extremely male-biased genes (fold change > 16 and *q* < 0.05) showed a striking enrichment of GO terms associated with sensory perception, detection of stimulus, and cell-cell signaling (*p* < 0.05, FDR-adjusted; Additional file 1: Supplementary Table 22). The significant activation of the sensory perception system of the adult males might be due to their short life (only about 2 days), while females usually live longer (about 20 days). Within the short lifespan of males, they need to find females to mate in a limited time frame; thus, they are reasonable to have a powerful sensory perception system. We identified 7 olfactory receptor genes with higher expression (21.14–415.75-fold higher) in the males than in the females. These genes might be related to locating the females for mating. The adult male has wings; however, the adult female is a wingless, ant-like wasp. Thus, as expected, a *flightin* gene that is involved in regulating flight muscle contraction, was 161.87-fold highly upregulated in the adult male than in the females (Additional file 1: Supplementary Table 23).

**Fig. 7:**
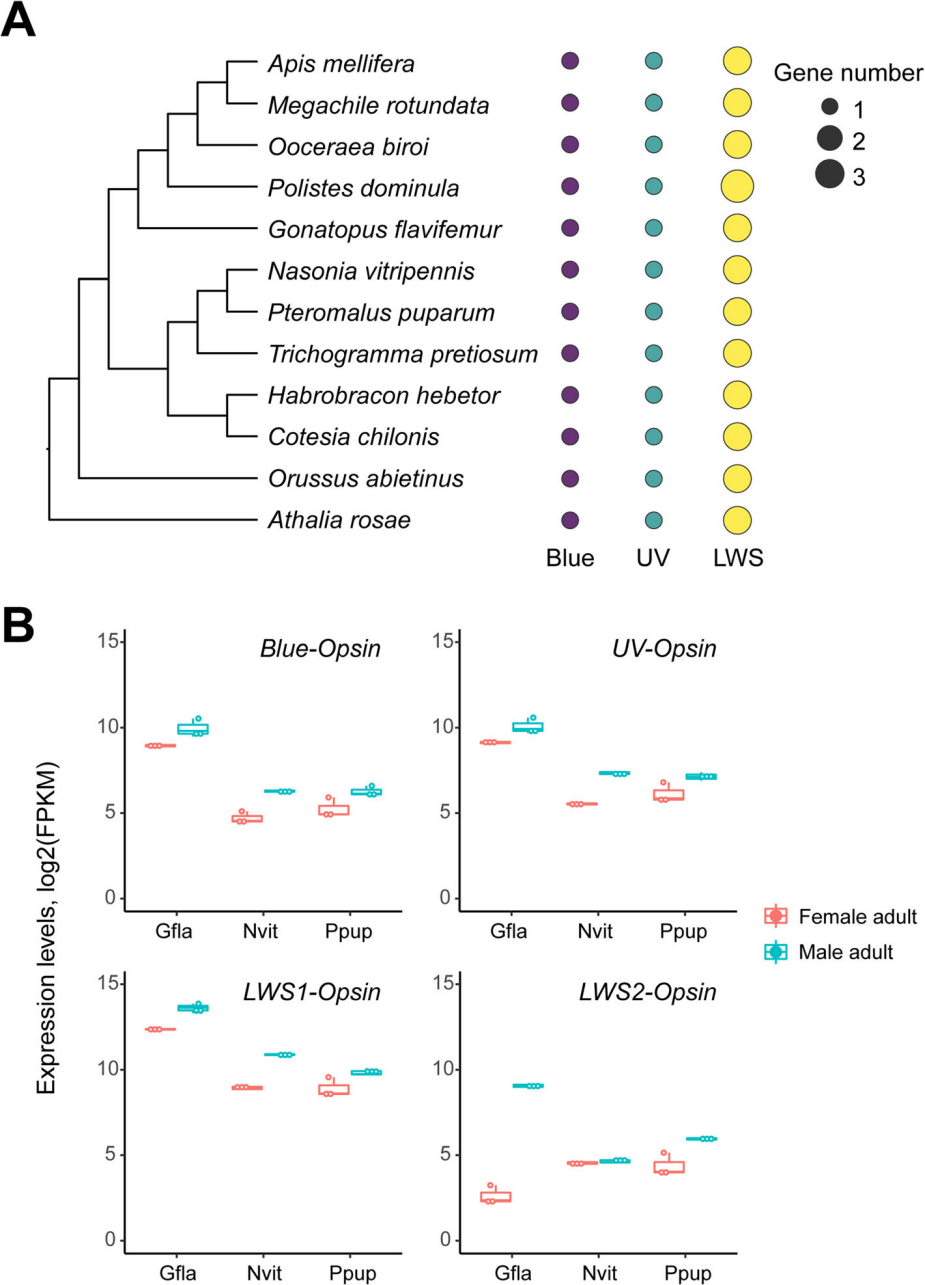
Opsin genes in *G. flavifemur.* **a** Opsin genes in the 12 hymenopteran insects. Bubble plot indicating the gene count of each opsin gene. Blue, short wavelength-sensitive opsin; UV, ultraviolet-sensitive opsin; LWS, long-wavelength-sensitive opsin. **b** Differential gene expression of opsin genes between female adult and male adult in three parasitoid wasps. The *Blue-opsin*, *UV-opsin* and *LWS1-opsin* in the three wasps are male-biased genes, with 1.89–4.02-fold higher expression in the males than in the females (*q* < 0.05). The *LWS2-opsin* of *G. flavifemur* was 86.86-fold higher male-biased expressed (*q* = 9.72e−106). The *LWS2-opsin* of *P. puparum* was 2.89 higher expressed in the males (*q* = 2.24e−06). However, the *LWS2-opsin* of *N. vitripennis* did not show any sex-biased expression pattern (*q* = 0.305). Gfla, *G. flavifemur*; Nvit, *N. vitripennis*; Ppup, *P. puparum*

We next analyzed the opsin genes, which are important in insect visual perception [98]. Our comparison among 12 hymenopteran insects showed that the ultraviolet (UV)-sensitive opsin and the blue-sensitive opsin are both kept as a single copy in all 12 hymenopterans. The paper wasp *Polistes dominula* has three long-wave sensitive (LWS) opsin genes, while the rest species have two LWS-opsin copies (Fig. 7a). Comparative transcriptome analysis among *G. flavifemur*, *P. puparum*, and *N. vitripennis* revealed that *Blue-opsin*, *UV-opsin*, and *LWS1-opsin* in the three wasps are male-biased genes, with 1.89–4.02-fold higher expression in the males than in the females (*q* < 0.05; Fig. 7b). Surprisingly, *LWS2-opsin* of *G. flavifemur* was 86.86-fold higher upregulated in the males compared to females (*q* = 9.72e−106), while the *LWS2-opsin* of *P. puparum* was only 2.89 higher expressed in the males (*q* = 2.24e−06). In addition, the *LWS2-opsin* of *N. vitripennis* did not show a male-biased expression pattern (*q* = 0.305; Fig. 7b). Taken together, our transcriptomic comparisons among *G. flavifemur*, *P. puparum*, and *N. vitripennis* indicate a global male-biased expression pattern of *Blue-opsin*, *UV-opsin*, and *LWS-opsin*, except the *LWS2-opsin* of *N. vitripennis*, and an extremely male-biased LWS2-opsin gene in *G. flavifemur*. The male-biased expression of these opsin genes may contribute to partner searching and mating.

## Discussion

Herein, by combining both Nanopore long-read sequencing and Illumina short-read sequencing strategies, we generated a high-quality reference genome of the pincer wasp *G. flavifemur*, which is the first genome sequence of the family Dryinidae. This species resides a key evolutionary position in Hymenoptera, as the early branch of the sting wasps (Aculeate), providing a valuable resource to facilitate our understanding of Hymenoptera evolution. In addition, *G. flavifemur* has many unique biological characteristics, including parasitism, predation, and sexual dimorphism.

In this study, our comparative genomics analysis highlighted a number of positively selected genes and rapidly evolving proteins involved in energy production and motor activity, which may play roles in the predatory adaptation of *G. flavifemur*. These findings expand our understanding of the hunting behavior evolution in Hymenoptera. By incorporating transcriptomic data, we found that 3 *yellow* genes (*yellow-g*, *yellow-g2* and *yellow-h*) were significantly activated in female *G. flavifemur* after feeding on the preys. It has been reported that some members of the *yellow* gene family are associated with reproductive maturation. For example, *yellow-g* and *yellow-g2* play a female-specific role in the egg development of *Drosophila* and *Aedes* [95, 96]. Moreover, all the *yellow* genes of silkworm *Bombyx mori* have a high transcription level in both ovary and testis, suggesting their reproduction-related functions [97]. Therefore, our discovery bridges the gap between the unique hunting behavior and reproductive advantages in the parasitoid wasp *G. flavifemur*.

Venoms are key evolutionary innovations in most hymenopteran insects and have been used for predatory, defensive, and reproductive purposes. Our comprehensive study on the venom gland identified significant expansions and unique expression patterns of *Idgf* genes and neprilysin-like genes. The functions of *Idgf* genes in insects are diverse, and they have been proved to participate in cuticle formation, wing development, larval and adult molting, immune response, antimicrobial response, and hemolymph clotting [88, 99, 100]. In addition, IDGF proteins are present in mosquito saliva and may contribute to the modulation of the mammalian host response and enhancing mosquito-borne Zika virus infection [101]. Here, we hypothesize that these IDGF proteins in the venom of *G. flavifemur* might play various potential roles, for example, regulating the development of the hosts, participating in the wound healing of the hosts, and immune-related functions against the hosts and bacteria. On the other hand, neprilysin specializes in the role of metabolizing and regulating molecules in the mammalian nervous systems; for example, it can inactivate peptide transmitters and their modulators [79, 102]. The expansion and venom high expression of neprilysin-like genes imply the *G. flavifemur* venom might have a powerful paralyzing effect on the host, which is of significant benefit to hunting and parasitism. Indeed, the temporary paralyzing effect of dryinid wasp’s venom has been reported before [3, 103], and our discovery of neprilysin expansion could explain the molecular basis of venom function. By comparing 31 hymenopterans, our study further indicated that venom neprilysins are prevalent in many hymenopterans and the recruitment events of venom neprilysin-like gene occurred independently during the radiation of Hymenoptera (i.e., convergent venom recruitments). However, this is only the tip of the iceberg, and exploring the bigger picture of venom neprilysin evolution in Hymenoptera requires further in-depth studies and whole-genome sequencing.

The genome size of *G. flavifemur* is much larger than most hymenopterans. The expansion of TEs, especially from DNA transposons, is a major contributor to the genome size enlargement in *G. flavifemur.* TE expansions and insertions can cause a variety of changes in the host genome, such as chromosomal rearrangements, gene disruptions, and gene expression regulations, some of which may be of benefit to adaptation [30, 104–107]. Several studies have shown that TE insertions play an essential role in helping insects to increase their adaptability, including raising the insecticide resistance and enhancing the ability to adapt to climate changes [108, 109]. In addition, TE is an important factor in insect antiviral immunity and aging regulation [110]. We therefore hypothesize that the expansion of TEs and the accompanied TE insertions might contribute to the adaptation of *G. flavifemur*. We envision further investigations on how TE expansion benefits the adaptation of *G. flavifemur*.

## Conclusions

Parasitoid wasps in the family Dryinidae display several interesting characteristics, such as the predatory behavior of adult females and the distinct sexual dimorphism. In this study, we present the genome of the dryinid wasp *G. flavifemur* to understand the genetic basis of these key innovations. Compared to other hymenopterans, our findings highlight that the TEs, especially DNA transposons, have massively expanded in the *G. flavifemur* genome, resulting in the genome size enlargement. Our genome-wide screens locate a number of positively selected genes and rapidly evolving proteins involved in energy production and motor activity, which may contribute to the predatory adaptation of *G. flavifemur*. We also show that 3 female-biased, reproductive-associated *yellow* genes in adult females expressed significantly higher after feeding on the preys, which may be beneficial to the egg production. This may explain the advantage of their unique predatory behavior. In addition, our transcriptomic analyses and following gene family analyses reveal the unique venom characters of *G. flavifemur*, such as the expansions of venom *Idgf*-like genes and neprilysin-like genes. Furthermore, we identify sex-biased genes based on the differences of gene expression between adult females and males, and observe an extremely male-biased LWS2-opsin gene in *G. flavifemur*. These results advance our understanding of the genome architecture, predatory adaptation, venom gene evolution, and sex-biased genes in *G. flavifemur*, and stimulate further comparative analyses of hymenopterans.

## Methods

### Insects

The dryinid wasps *G. flavifemur* were provided by Dr. Qiang Fu from his laboratory. The wasp colony was originated from about 50 wasps of a field population, which was collected at Hangzhou, China, in 2018. The colony of the hosts *N. lugens* was first collected from rice fields at Hangzhou, China, in 2018. Both parasitoid wasps and the hosts were maintained in the laboratory. The *N. lugens* was reared continuously on a susceptible rice variety (Taichung Native 1, TN1) under laboratory conditions at 28 ± 1 °C, 65 ± 5% relative humidity (RH), 3500 ~ 4000 Lux and a photoperiod of 16: 8 h (light: dark) [111]. The parasitoid wasps *G. flavifemur* were reared on 4–5th instar brown planthoppers under the same conditions. The details of samples for genome and transcriptome sequencing are summarized in Additional file 1: Supplementary Table 24.

### Genome sequencing

We applied the Nanopore and Illumina HiSeq X Ten platforms to sequence the genome of *G. flavifemur.* High-quality genomic DNA for de novo sequencing was extracted from 50 haploid male pupae using the sodium dodecyl sulfate (SDS)-based DNA extraction method followed by purification with VAHTS DNA Clean Beads (Vazyme, Cat. # N411-03) according to the standard procedure provided by the manufacturer. The quality and concentration were then assessed by 1% agarose gel electrophoresis, NanoDrop™ One UV-Vis spectrophotometer (Thermo Fisher Scientific, USA) and Qubit® 3.0 Fluorometer (Invitrogen, USA). For long library preparation, qualified DNA was size-selected (> 10 kb) using the BluePippin system (Sage Science, USA), then two long libraries were processed according to the Ligation Sequencing Kit (SQK-LSK109, Oxford Nanopore) protocol and sequenced on two flow-cells using the PromethION sequencer (Oxford Nanopore). A short paired-end library with an insert size of 350 bp was constructed using a TruSeq Nano DNA HT Sample Preparation Kit (Illumina), and then sequenced on the HiSeq X Ten platform.

### Evaluation of genome size

The paired-end Illumina reads were firstly filtered by fastp v0.20.0 [112]. Clean reads were used for estimating the genome size and heterozygosity using GenomeScope v1.0.0 [113] based on the 17-mer distribution analyzed by Jellyfish v2.3.0-1 [114]. The estimated genome size was further validated by flow cytometry following the standard procedure reported in He et al. [115]. Briefly, the heads of 20 adult insects were completely homogenized in 500 μL ice-cold Galbraith’s Buffer (45 mM MgCl_2_, 20 mM 3-N-morpholinopropane sulfonic acid, 30 mM sodium citrate, and 0.1% (vol/vol) Triton X-100; pH 7.0). The homogenate was then filtered into a 1.5-mL Eppendorf tube using 38-μm nylon mesh. To remove RNAs, RNase A (Takara, Japan) was added to the homogenate (final concentration of 20 μg/mL) and incubated at 25 °C for 10 min. The precipitates were collected by centrifuging at 1000*g* for 5 min, and then suspended with 400 μL phosphate buffer (pH 7.4) and stained with 50 μg/mL propidium iodide stock solution in darkness at 4 °C for 10 min. Each sample was analyzed using the MoFlo™ XDP High Speed Cell Sorter and Analyzer (Beckman Coulter, CA, USA) under 488-nm wavelength. Summit Software (Beckman Coulter, CA, USA) was used to obtain the nuclei peaks. The genome size was then estimated based on the outputs, using *D. melanogaster* as a control (Additional file 2: Supplementary Figure 2).

### Genome assembly and assessment

We firstly used NextDenovo v1.1.1 (https://github.com/Nextomics/NextDenovo) to correct Nanopore reads with -seed_cutoff = 10 k parameter. The corrected reads were then assembled into primary contigs using wtdbg2 v2.3 [116]. Then, NextPolish v1.0.5 [117] was used to polish the assembly with both Nanopore long reads (three iterations) and Illumina reads (four iterations). Finally, we removed the bacterial contaminating contigs using the pipeline of Wheeler et al. [11]. Briefly, each contig was firstly split into 1000 bp units, which were then searched against a bacterial genome database provided in Olafson et al. [15] using BLASTN v2.8.1 [16] (-evalue 1e−5). Contigs were identified as likely bacterial contigs if the proportion of bacterial matched units along their total number of units was larger than 40%. BUSCO v5 [118] was used to assess the genome assembly completeness with the insect protein set (insecta_odb10). We also mapped the Illumina genomic reads and RNA-seq reads to the genome assembly by BWA v0.7.17 [119] and HISAT2 v2.2.1 [120] respectively. The mapping rates were counted by SAMtools v1.10 [121].

### Transcriptome sequencing and analysis

RNA-seq libraries (insert size of 250 bp) were prepared from larva (7-day-old larvae, 10 individuals), pupa (10 individuals), female adult (1-day-old, 10 individuals), male adult (1-day-old, 10 individuals), venom gland (5-day-old female adult, 100 individuals), and carcass (5-day-old female adult, remove venom gland, 10 individuals). In addition, to investigate the gene expression changes between prey-feeding females and none prey-feeding females, six RNA-seq libraries (insert size of 250 bp) were prepared from the female adults (5-day-old) reared on 20% sucrose water and brown planthoppers (the preys), respectively. Three biological replicates were prepared for each sample. The transcriptomes were sequenced using the Illumina HiSeq X Ten platform with paired-end libraries. Raw reads from the RNA-seq were filtered using Trimmomatic v0.38 [122]. The clean reads were mapped to the genome assembly using HISAT2 v2.2.1 [120] and then assembled into transcripts using StringTie v2.1.0 [123]. RSEM v1.3.3 [124] was used for estimating the gene expression level, and DESeq2 package v1.30.1 [125] was used to perform the differential expression analyses.

### Repeat annotation

We used the Extensive de novo TE Annotator (EDTA) pipeline to construct TE libraries for each species [126]. Briefly, we firstly applied a series of structure-based TE classification tools in the EDTA pipeline to identify each type of TE (LTR_FINDER [127], *LTRharvest* [128] and LTR_retriever [129] for LTR retrotransposons, TIR-Learner [130] and HelitronScanner [131] for DNA transposons). Outputs from each program were filtered by the EDTA pipeline (the filtering step was detailly described in Ou et al. [126]). Next, RepeatModeler v2.0 [132] was used to identify non-LTR retrotransposons and any unclassified TEs that were missed by the TE annotators above. Finally, all results were compiled into a comprehensive non-redundant TE library for downstream analysis. RepeatMasker v4.0.7 [133] was then used to search for known and novel TEs by mapping sequences against the de novo TE library and Repbase library v16.02 [134]. Tandem repeats were annotated using Tandem Repeat Finder v4.09 [135]. The divergences of each TE family were reported by RepeatMasker and then converted to nucleotide distance measures using the Jukes-Cantor nucleotide model to correct for multiple hits. Final results were pooled into bins of single unit distances, which recapitulates the history of TE class proliferation.

### Gene annotation

Three approaches, as incorporated in the EVidenceModeler pipeline (EVM, v1.1.1) [136], were used to predict the protein-coding genes: de novo gene prediction, homology-based and RNA-seq-based approaches. For de novo gene prediction, we utilized AUGUSTUS v3.1 [137] and SNAP v2006-07-28 [138] to analyze the repeat-masked genome. For homology-based predictions, the protein sequences of invertebrates were retrieved from NCBI Reference Sequence Database as templates. Exonerate v2.2.0 (https://www.ebi.ac.uk/about/vertebrate-genomics/software/exonerate) and GenomeThreader v1.7.1 [139] were used to align the reference proteins to the genome assembly and predict gene structures. For RNA-seq-based gene prediction, we used the transcriptome assembled from RNA-seq alignments and identified the candidate coding region of each transcript by TransDecoder v5.5.0 (https://github.com/TransDecoder/TransDecoder). Finally, EVidenceModeler v1.1.1 [136] was used to integrate the genes predicted by the above three approaches and generate a consensus gene set. Gene Ontology (GO) analysis was carried out using the software Blast2GO v5.2 [140]. We next mapped the gene set to Kyoto Encyclopedia of Genes and Genomes (KEGG) pathways using BlastKOALA v2.2 [141] online service. We also searched the protein sequences in the SwissProt and TrEMBL databases using BLASTP v2.8.1 [16] (-evalue 1e−5).

### Comparative genomics

We comprehensively considered three factors of each species for comparative genomics analyses and phylogenomic analyses, including genome quality, popularity, and evolutionary position. OrthoFinder v2.5.1 [20] was used to identify the orthologous and paralogous genes of 13 Hymenoptera genomes including *O. abietinus* (RefSeq assembly accession: GCF_000612105.2), *C. chilonis* (http://www.insect-genome.com/), *Habrobracon hebetor* (Ye et al. [142]), *Trichogramma pertiosum* (RefSeq assembly accession: GCF_000599845.2), *Nasonia vitripennis* (OGS2.0, Rago et al. [143]), *P. puparum* (OGS1.0, Ye et al. [13]), *G. flavifemur* (this study), *P. dominula* (RefSeq assembly accession: GCF_001465965.1), *Solenopsis invicta* (RefSeq assembly accession: GCF_000188075.2), *Ooceraea biroi* (RefSeq assembly accession: GCF_003672135.1), *Megachile rotundata* (RefSeq assembly accession: GCF_000220905.1), *A. mellifera* (RefSeq assembly accession: GCF_003254395.2), and *A. rosae* (RefSeq assembly accession: GCF_000344095.2). The basal hymenopteran *A. rosae* was used as an outgroup.

### Phylogenetic analysis

A total of 2992 one-to-one orthogroups shared by the 13 Hymenoptera species were extracted for phylogenetic analysis. The protein sequences in each orthogroup were independently aligned by MAFFT v7 [21] and filtered by trimAl v1.2 [22] with the default parameters. These sequences were concatenated to generate a supergene sequence, which was used for tree construction. The phylogenetic tree was constructed by maximum likelihood (ML) using IQ-TREE v2.1.2 [23] with the best model (LG + I + G) estimated by ModelFinder [24]. Statistical support for the phylogenetic trees was assessed by Ultrafast bootstrap [25] analysis using 1000 replicates. The standard concatenation approaches do not model discordance among gene trees beyond differences in sequence evolution rates [144]. Many studies have shown that incomplete lineage sorting (ILS) has the potential to lead to incorrect topology, possibly due to the estimation bias in a concatenated analysis where the mixture of gene trees represents a model violation [145]. To overcome these limitations, ASTRAL-III [146], a multispecies coalescent tool, was used to summarize all the 2992 gene trees and measure branch supports as local posterior probabilities. Both concatenation and multispecies coalescent approach yield the same topology species tree. The MCMCtree program in the PAML package v4.9e [147] was used to estimate divergence time based on protein sequences. Five calibration time points based on a previous study were used, Orussoidea+Apocrita: 211–289 million years ago (mya), Apocrita: 203–276 mya, Ichneumonoidea: 151–218 mya, Chalcidoidea: 105–159 mya, Aculeata: 160–224 mya [2].

### Correlation between genome size and TE content

Considering the phylogenetic relationships of the species studied, we first used the R caper package v1.0.1 (https://CRAN.R-project.org/package = caper) to test the effect of the phylogeny on the correlation between genome size and TE content. We obtained a lambda parameter from 0 to 1 (0 indicates covariances between taxa are negligible in these clades and thus those correlations are not biased by the phylogeny, and 1 means the evolutionary relationships among surveyed species induced a bias in the correlation calculation) by fitting a linear model on the data. We then computed corrected Pearson’s correlation between the genome size and the TE content using the ape package v5.4-1 [148] in R if evolutionary relationships induced a bias in the correlation calculation.

### Identification of positively selected genes

We used two packages, PAML v4.9 [147] and HyPhy [149] to detect positive selection signals on the *G. flavifemur* branch. In total, 2992 single-copy gene families were used for positive selection analyses. For PAML analysis, these single-copy genes were detected using the optimized branch-site model. A likelihood ratio test (LRT) was conducted to compare the null model (sites under neutrally and under purifying selection) and the alternative model (sites under positive selection on the foreground branch). The p values were computed based on chi-square statistics with a false discovery rate (FDR), and genes with p-adjusted value less than 0.05 were identified as positive selection genes. For HyPhy analysis, the aBSREL algorithm [150] was used for positive selection signal searching. Genes with test *p* values < 0.05 were considered to be under positive selection. Finally, 183 genes detected by both methods were treated as candidates that underwent positive selection.

### Identification of rapidly evolving proteins

Based on 2992 single-copy proteins, we used a rank-based branch length comparison method to identify rapidly evolving proteins on *G. flavifemur* branch as used in the *Trichogramma* genome [14]. In brief, a phylogenetic tree of each single-copy orthogroup was reconstructed using IQ-TREE v2.1.2 [23]. We then compared the rank of total branch length and the rank of *G. flavifemur* branch length. To avoid overrepresentation among any protein category based on general evolutionary rates, we next binned proteins into groups of 300 based on the total branch length rank. Rapidly evolving proteins were defined as proteins with the top 10% largest values of the *G. flavifemur* branch length rank minus the total branch length rank in each group.

### Gene family expansion and contraction

We used CAFE v4.2.1 [151] to analysis the gene family expansion and contraction. The results from OrthoFinder and a phylogenetic tree with divergence times were used as inputs.

### Identification of venom gland-associated genes in *G. flavifemur*

To identify the venom gland-associated genes, we firstly calculated the expression level of each transcript in the venom gland and the carcass (i.e., adult female tissues minus the venom gland) using RSEM v1.3.1 [124]. To qualify a gene as a venom gland-associated gene, it must be (a) among the top 500 expressed genes in the venom gland transcriptome, and (b) highly expressed in the venom gland relative to the carcass (*q* < 0.05), and (c) not highly expressed in the carcass (median FPKM < 50). The signal peptide of each venom associate gene was predicted using the SignalP v5.0b [152].

### Identification of genes involved in detoxification and chemosensory

To identify genes involved in detoxification and chemosensory, cytochrome P450s, glutathione S-transferases (GSTs), ATP-binding cassette transporters (ABCs), gustatory receptors (GRs), ionotropic receptors (IRs), olfactory receptors (ORs), odorant binding proteins (OBPs), sensory neuron membrane proteins (SNMPs), and chemosensory proteins (CSPs) protein sequences of well-annotated insects retrieved from Uniprot were used as queries to search against the predicted protein sequences from *G. flavifemur* and other 12 hymenopteran genomes mentioned in the comparative genomics section using BLASTP v2.8.1 [15] (-evalue 1e−5). All candidate detoxification and chemosensory genes were further checked for the presence of their characteristic domains to confirm their identity, P450s: PF00067, GSTs: PF00043 or PF02798, ABCs: PF00005, GRs: PF08395 or PF06151, IRs: PF00060, ORs: PF02949 or PF13853, OBPs: PF01395, SNMPs: PF01130, CSPs: PF03392.

### Chitinase-like genes

To annotate the GH-18 chitinase-like genes, a number of well-annotated insect chitinase protein sequences were used as queries in a BLASTP search (-evalue 1e−5) against the proteins of 17 insect genomes including 13 hymenopteran genomes mentioned in the comparative genomics section, *Bombyx mori* (RefSeq accession: GCF_014905235.1), *Tribolium castaneum* (RefSeq accession: GCF_000002335.3), *Drosophila melanogaster* (RefSeq accession: GCF_000001215.4), and *Acyrthosiphon pisum* (RefSeq accession: GCF_005508785.1). We next checked the putative chitinase-like genes containing the glycosyl hydrolase 18 (GH-18) domain using hidden Markov models (HMM search) [153]. Finally, we manually confirmed the alignments of the GH18 domains and removed the sequences if they were only partially aligned. A HMM searching against the Pfam-A database [154] was then performed using the candidate chitinase-like proteins to identify additional domains (i.e., carbohydrate binding domains). Phylogenetic analysis of the chitinase-like proteins was performed using maximum likelihood methods with LG + R5 model (all chitinase-like proteins) and LG + G4 model (group 5 chitinase-like proteins) determined by ModelFinder [24] in IQ-TREE v2.1.2 [23]. Statistical support for all phylogenetic trees was assessed by Ultrafast bootstrap [25] analysis using 1000 replicates. Chitinase-like proteins were classified into subgroups based on the domain architecture and phylogenetic analysis as summarized in Arakane and Muthukrishnan [88]. The conserved catalytic domain in insect chitinase [86, 88, 155] was searched using the candidate chitinase-like proteins of *G. flavifemur* based on the multiple sequence alignment generated by MAFFT v 7.471 [21] with L-INS-I model.

### Neprilysin-like genes

To identify neprilysin-like genes, we retrieved the well-annotated neprilysin sequences from UniProtKB/SwissProt as queries. BLASTP (-evalue 1e−5) was performed for protein searching in the 17 insect genomes described in chitinase-like gene section. Each protein sequence obtained was subsequently used for searching against Pfam-A [154] database by HMMscan v3.3.2 [153] (--cut_ga) to confirm the presence of the Peptidase_M13 domain. Candidate neprilysin-like proteins were manually checked for the alignments of Peptidase_M13 domain. To gain insights into the venom neprilysin-like genes of other hymenopteran species, we annotated the neprilysin-like genes in the venom genes of additional 30 hymenopteran insects (15 in Parasitoida and 15 in Aculeate) using the same method [35, 67–70, 75, 77, 89, 90, 156–164]. The phylogeny of the hymenopterans in Fig. 5c was obtained from previous studies [2, 91]. Phylogenetic analysis of the neprilysin-like genes was performed using maximum likelihood methods using LG + R9 model determined by ModelFinder [24] in IQ-TREE v2.1.2 [23]. Statistical support for all phylogenetic trees was assessed by Ultrafast bootstrap [25] analysis using 1000 replicates.

### Identification of opsin genes

Amino acid sequences of opsin genes in *A. mellifera*, *D. melanogaster*, and *T. castaneum* were obtained from UniProt. They were used for searching the proteins against 13 hymenopteran genomes using BLASTP v2.8.1 [16] (-evalue 1e−10). To discriminate opsins from other G-protein-coupled receptors (GPCRs), we used a combination of sequence similarity and motif analysis described in Feuda et al. [98]. Briefly, an opsin gene should have a top BLASTP hit with opsin in Uniprot and/or contain a recognizable retinal-binding domain.

### Enrichment analysis

GO enrichment analyses were conducted by GOATOOLS v1.0.6 [165], a python library for gene ontology analysis.

## Supplementary Information

**Additional file 1: Supplementary Table 1.** Statistics of Nanopore sequencing. **Supplementary Table 2.** Statistics of Illumina sequencing. **Supplementary Table 3.** Statistics of *Gonatopus flavifemur* genome assembly by Nanopore reads. **Supplementary Table 4.** Statistics of *Gonatopus flavifemur* genome assembly after error-corrected by Illumina and Nanopore reads. **Supplementary Table 5.** Genome size of *G. flavifemur* estimated by flow cytometry and 17-mer analysis. **Supplementary Table 6.** The mapping rate with short sequencing reads of *Gonatopus flavifemur* genome. **Supplementary Table 7**. Genes in detoxification system and chemoreception system in 13 hymenopterans. **Supplementary Table 8.** TE sequences in *Gonatopus flavifemur* and other surveyed species. **Supplementary Table 9.** Characteristics of DNA TEs in *Gonatopus flavifemur*. **Supplementary Table 10.** GO enrichment analysis of Aculeate-specific genes, Biological Process category (padj < 0.05). **Supplementary Table 11.** GO enrichment analysis of *Gonatopus flavifemur* special genes. (padj < 0.05). **Supplementary Table 12.** Genes under positive selection in *Gonatopus flavifemur* detected by both aBSREL model in HyPhy and branch-site model in PAML. **Supplementary Table 13.** GO enrichment analysis of *Gonatopus flavifemur* expanded gene families, Biological Process category (padj < 0.05). **Supplementary Table 14.** Venom gland-associated genes (VGGs) in *Gonatopus flavifemur*. **Supplementary Table 15.** GO enrichment analysis of VGGs in *Gonatopus flavifemur*, Biological Process category (padj < 0.05). **Supplementary Table 16.** Chitinase-like genes in *Gonatopus flavifemur* and other species. **Supplementary Table 17.** Neprilysin-like genes in *Gonatopus flavifemur* and other Hymenoptera genomes. **Supplementary Table 18.** Upregulated genes in the prey-feeding female transcriptomes compared to the sucrose-feeding female transcriptomes. **Supplementary Table 19.** *Yellow* genes and expression level in *Gonatopus flavifemur*. **Supplementary Table 20.** Downregulated genes in the prey-feeding female transcriptomes compared to the sucrose-feeding female transcriptomes. **Supplementary Table 21.** GO enrichment analysis of extremely female-biased genes in *Gonatopus flavifemur*. Biological Process category (padj < 0.05). **Supplementary Table 22.** GO enrichment analysis of extremely male-biased genes in *Gonatopus flavifemur*. Biological Process category (padj < 0.05). **Supplementary Table 23.** Extremely male-biased genes in *Gonatopus flavifemur*. **Supplementary Table 24.** Sample information for sequencing used in this study.

**Additional file 2: Supplementary Figure 1.** Genome size of *G. flavifemur* estimated by k-mer analysis. **Supplementary Figure 2.** Flow cytometry histograms of *Drosophila melanogaster* (A) and *G. flavifemur* (B). **Supplementary Figure 3.** Concatenated- and ASTRAL-based phylogenetic trees. **Supplementary Figure 4.** Rank-based branch length comparison of protein evolution rates in the *G. flavifemur* branch. **Supplementary Figure 5.** Venom gland-associated genes (VGGs) in *G. flavifemur.* **Supplementary Figure 6.** Multiple sequence alignment of the active site motif (DxxDxDxE) of IDGFs in *G. flavifemur* and other 12 hymenopterans.

**Additional file 3. **Bacterial contaminating contigs in the *G. flavifemur* genome assembly.

## Data Availability

All the data generated in this study are available at the National Center for Biotechnology Information (NCBI), under BioProject number PRJNA695321 [166]. The genome assembly has been deposited at GenBank under accession JAFFJZ000000000 [167]. The genome and transcriptome sequencing data have been deposited in the NCBI Sequence Read Archive (SRA) database (SRR14374062, SRR14374063 and SRR13625245-SRR13625267). In addition, all the sequencing data and annotation information are available in InsectBase (http://insect-genome.com/Gfla/) [168].

## Abbreviations

IDGF: Imaginal disc growth factor
LWS: Long-wave sensitive
GSTs: Glutathione S-transferases
ABCs: ATP-binding cassette transporters
GRs: Gustatory receptors
IRs: Ionotropic receptors
ORs: Olfactory receptors
OBPs: Odorant binding proteins
SNMPs: Sensory neuron membrane proteins
CSPs: Chemosensory proteins
BUSCO: Benchmarking universal single-copy orthologs
NCBI: National Center for Biotechnology Information
TEs: Transposable elements
LTRs: Long terminal repeat retrotransposons
LINEs: Long interspersed nuclear elements
SINEs: Short interspersed nuclear elements
MITEs: Miniature inverted-repeat transposable elements
GO: Gene Ontology
FDR: False discovery rate
NDUFB3: NADH dehydrogenase ubiquinone 1 beta subcomplex subunit 3
NDUFA9: NADH dehydrogenase ubiquinone 1 alpha subcomplex subunit 9
mtTFB1: Mitochondrial transcription factor B1
PTCD3: Pentatricopeptide repeat domain-containing protein 3
TLN1: Talin-1
TBCE: Tubulin-specific chaperone E
Scgβ: Beta-sarcoglycan
SPG11: Spatacsin 11
ALS2: Amyotrophic lateral sclerosis 2
TWF1: Twinfilin 1
SIM: Single-minded
AGO3: Argonaute 3
ETFRF1: Electron transfer flavoprotein regulatory factor 1
NDUFB7: NADH dehydrogenase ubiquinone 1 beta subcomplex subunit 7
cGK: cGMP-dependent protein kinase
GTF2IRD: General transcription factor II-I repeat domain-containing
VGGs: Venom gland-associated genes
FPKM: Fragments per kilobase per million mapped fragments
GH18: Glycoside hydrolase 18
UV: Ultraviolet
SDS: Sodium dodecyl sulfate
KEGG: Kyoto Encyclopedia of Genes and Genomes
Mya: Million years ago
LRT: Likelihood ratio test
HMM: Hidden Markov models
