# Supplementary material for: Genome of the pincer wasp Gonatopus flavifemur reveals unique venom evolution and a dual adaptation to parasitism and predation
Source: BMC Biol. 2021 Jul 27;19:145. doi: 10.1186/s12915-021-01081-6 (PMC8314478; doi:10.1186/s12915-021-01081-6)
Supplement: Supplementary file 2 — Additional file 2: Supplementary Figure 1. Genome size of G. flavifemur estimated by k-mer analysis. Supplementary Figure 2. Flow cytometry histograms of Drosophila melanogaster (A) and G. flavifemur (B). Supplementary Figure 3. Concatenated- and ASTRAL-based phylogenetic trees. Supplementary Figure 4. Rank-based branch length comparison of protein evolution rates in the G. flavifemur branch. Supplementary Figure 5. Venom gland-associated genes (VGGs) in G. flavifemur. Supplementary Figure 6. Multiple sequence alignment of the active site motif (DxxDxDxE) of IDGFs in G. flavifemur and other 12 hymenopterans. [file 12915_2021_1081_MOESM2_ESM.docx]

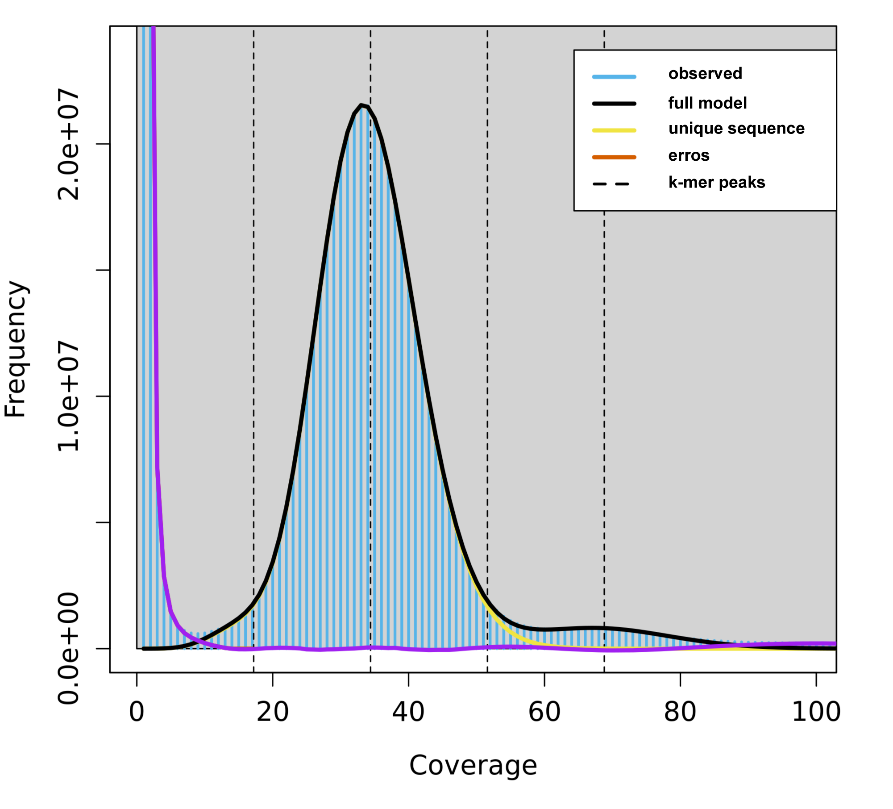


Supplementary Figure 1. Genome size of *G. flavifemur* estimated by k-mer analysis. The k-mer (k= 17) frequency profile was generated by GenomeScope v1.0.0, showing an estimated genome size of 603.4 Mb.


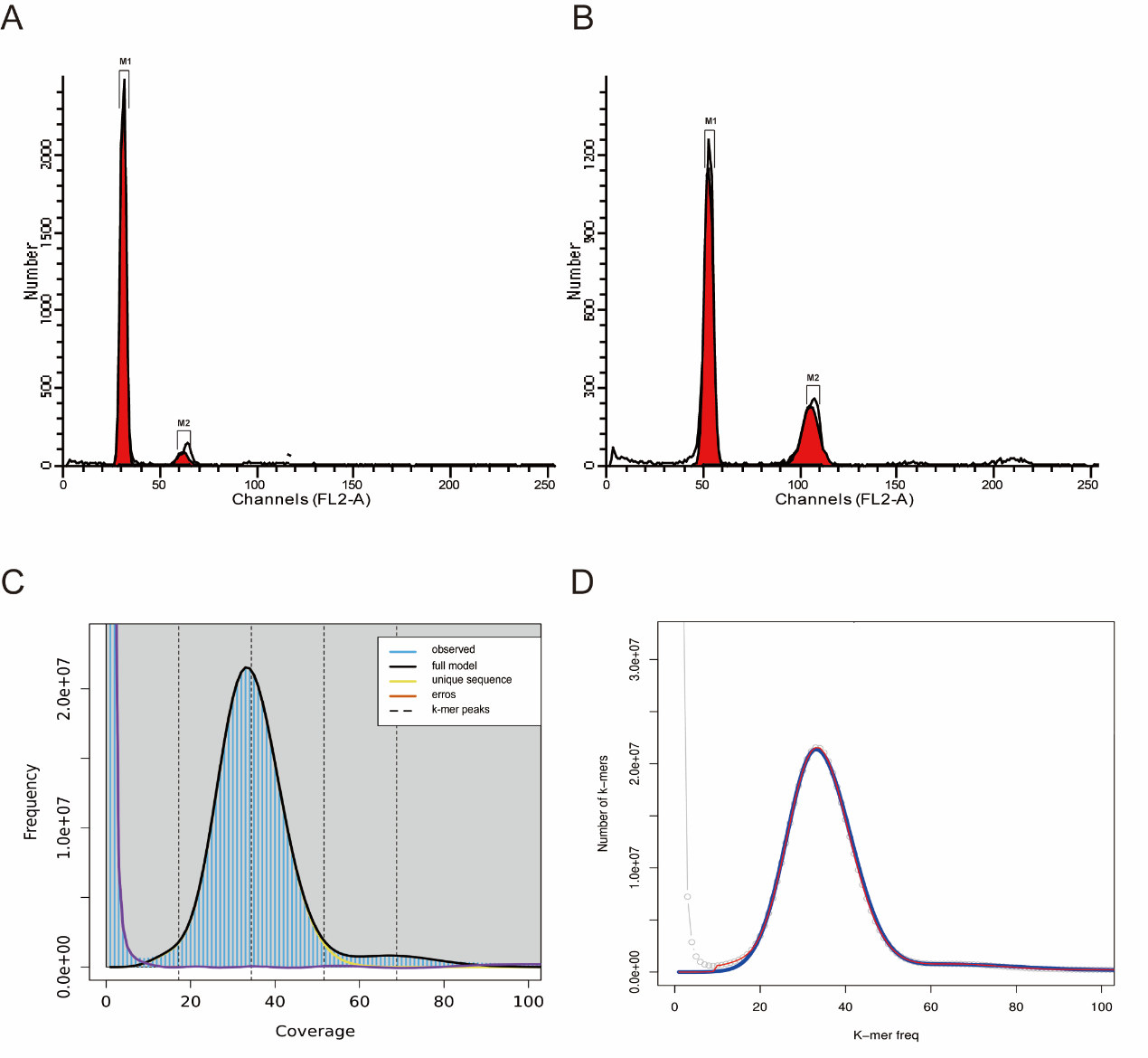
Supplementary Figure 2. Flow cytometry histograms of *Drosophila melanogaster* (A) and *G. flavifemur* (B). The genome size of *G. flavifemur* (B, M1) was estimated at 601.4 Mb in comparison with *D. melanogaster* (A, M1).


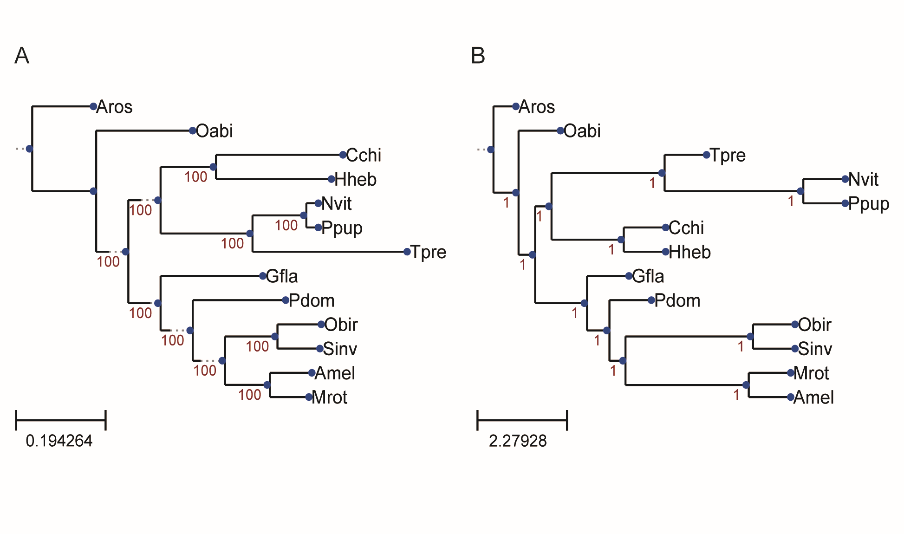


Supplementary Figure 3. Concatenated- and ASTRAL-based phylogenetic trees. (A) Phylogenetic trees based on the 2,992 concatenated single-copy orthologous groups from *G. flavifemur* and other 12 hymenopterans using IQ-TREE. (B) Phylogenetic trees based on multi-species coalescent (MSC) methods based on the 2,992 single-copy orthologous from *G. flavifemur* and other 12 hymenopterans using ASTRAL-III. Aros: *A. rosae*; Oabi: *O. abietinus*; Cchi: *C. chilonis*; Hheb, *H. hebetor*, Nvit: *N. vitripennis*; Ppup, *P. puparum*; Tpre: *T. pertiosum*; Gfla, *G. flavifemur*; Pdom: *P. dominula*; Obir: *O. biroi*; Sinv: *S. invicta*; Amel: *A. mellifera*; Mrot: *M. rotundata*. The basal hymenopteran *A. rosae* was used as an outgroup.


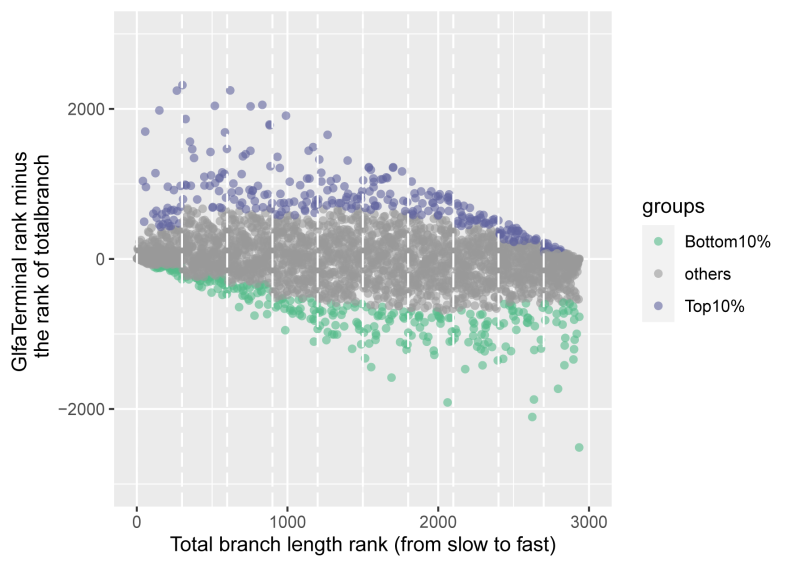


Supplementary Figure 4. Rank-based branch length comparison of protein evolution rates in the *G. flavifemur* branch.


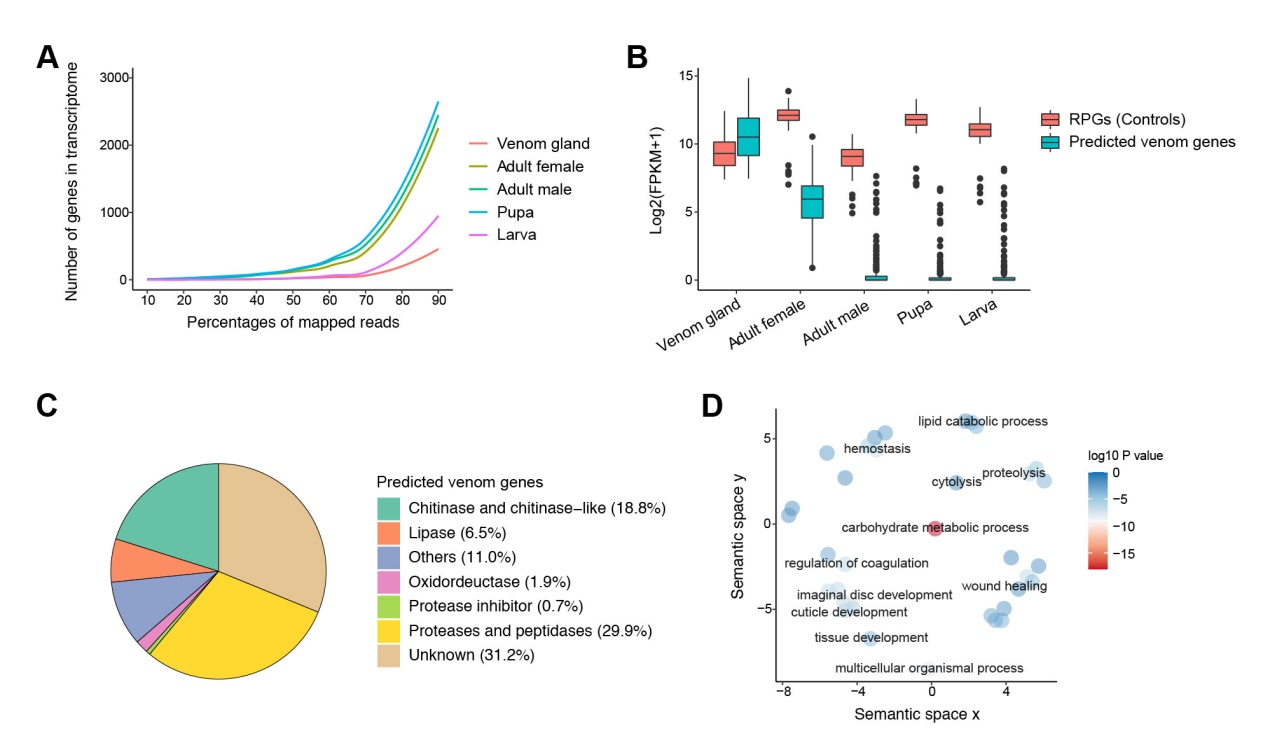


Supplementary Figure 5. Venom gland-associated genes (VGGs) in *G. flavifemur.* (A) The number of genes that account for different percentages of mapped reads in the venom gland (red), adult female (brown), adult male (green), pupa (blue) and larva (purple) transcriptomes of *G. flavifemur*, showing gene expression specialization in the venom gland. (B) Expression of VGGs and RPGs in different developmental stages of *G. flavifemur.* (C) Categories of the 154 VGGs in *G. flavifemur.* (D) GO enrichment analysis of VGGs in *G. flavifemur.*


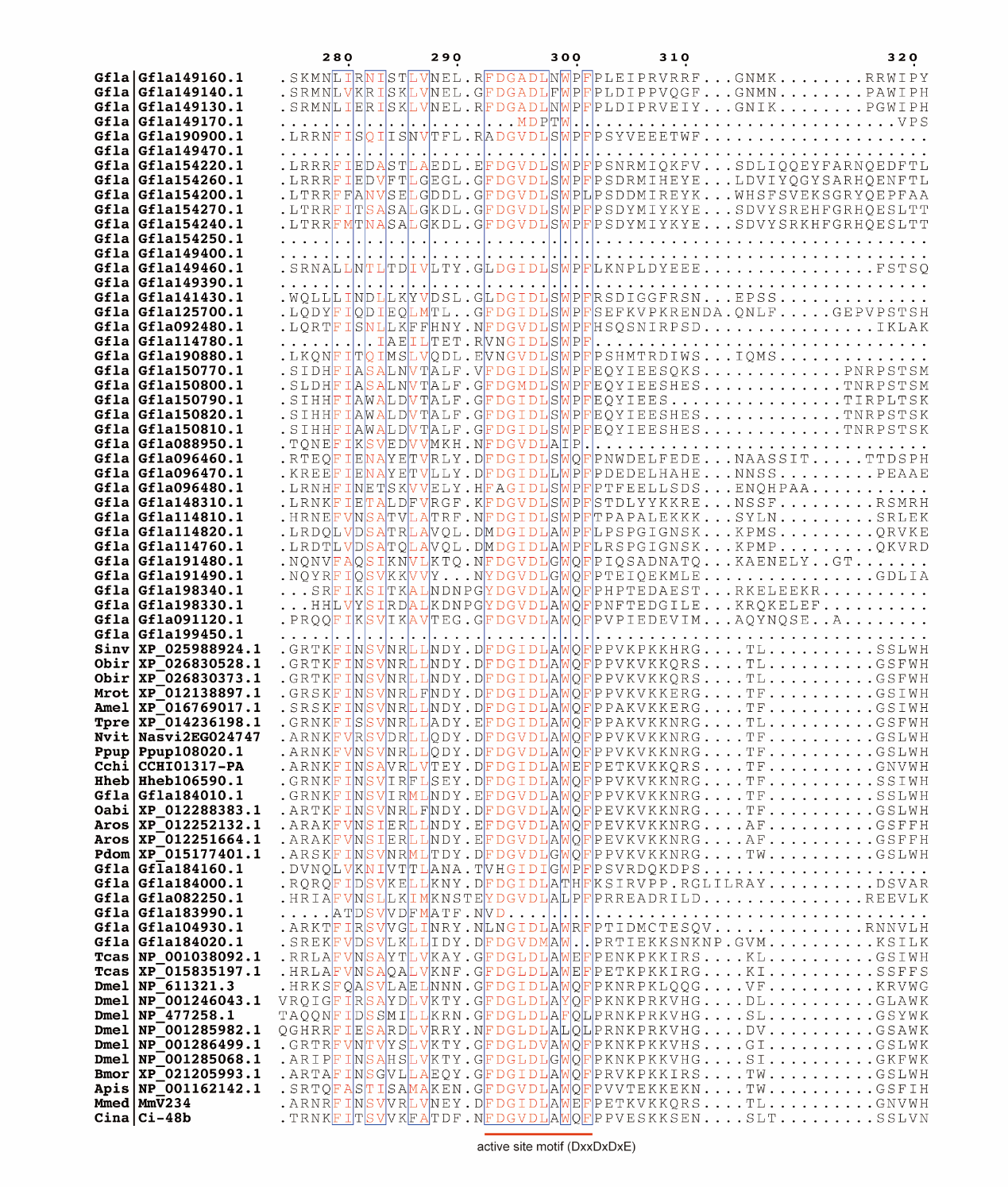


Supplementary Figure 6. Multiple sequence alignment of the active site motif (DxxDxDxE) of IDGFs in *G. flavifemur* and other 12 hymenopterans. The active site motif (DxxDxDxE) is indicated by red line.
