# Supplementary material for: Genome of the pincer wasp Gonatopus flavifemur reveals unique venom evolution and a dual adaptation to parasitism and predation
Source: BMC Biol. 2021 Jul 27;19:145. doi: 10.1186/s12915-021-01081-6 (PMC8314478; doi:10.1186/s12915-021-01081-6)
Supplement: Supplementary file 3 — Additional file 3. Bacterial contaminating contigs in the G. flavifemur genome assembly. [file 12915_2021_1081_MOESM3_ESM.docx]

**Additional File 3: Bacterial contaminating contigs in the *G. flavifemur* genome assembly.**

**Methods**

**DNA-based pipeline for identification of the bacterial contaminating contigs in the *G. flavifemur* genome assembly**

The identification pipeline of the bacterial contaminating contigs was firstly developed by David Wheeler and John H. Werren [11, 12] and has been used for bacterial contamination identification in a number of genome sequencing projects [12–14]. We used this pipeline for bacterial contamination identification in the *G. flavifemur* genome assembly. Briefly, each contig of the genome assembly was firstly split into 1,000 bp units, which were then searched against a bacterial genome database provided in Olafson et al. [15] using BLASTN (-evalue 1e-5) [16]. Contigs were identified as likely bacterial contigs if the proportion of bacterial matched units along their total number of units was larger than 40%.

**Annotation of *wGfla***

The gene model prediction and functional annotation of *wGfla* genome were performed by the RAST [17] genome annotation service and eggNOG-mapper v2 [18] with default parameters. 16s rDNA predicted by RAST was then searched against the NT database using BLASTN [16]. Whole genome alignment was performed using the online service for comparative genomic analysis, D-GENES [19].

**Phylogenetic analysis**

To assess the phylogenetic relationships of *wGfla*, we inferred phylogeny using the protein-coding sequences from all available genomes with high quality annotations of *Wolbachia* from NCBI/Uniprot (Table 1). Three Anaplasmataceae genomes (Table 1) were used as outgroup. Briefly, OrthoFinder v2.5.1 [20] was used to identify the universe single copy genes in the *Wolbachia* genomes and Anaplasmataceae genomes. In total, 192 single copy genes were identified and then aligned by MAFFT v7 [21], filtered by trimAl v1.2 [22] with the default parameters. These sequences were concatenated to generate a supergene sequence, which was used for tree construction. A maximum likelihood (ML) tree was inferred using IQ-TREE v2.1.2 [23] with the best model (JTT + F + R4) estimated by ModelFinder [24]. Statistical support for the phylogenetic tree was assessed by Ultrafast [25] bootstrap analysis using 1,000 replicates.

**Results**

Many arthropod genome assemblies contained bacterial genomes from symbiotic bacteria or bacterial contamination during sample preparation or genome sequencing [2–4]. To investigate the bacterial contigs in the *G. flavifemur* genome assembly, we used a pipeline to identify likely bacterial contigs in *G. flavifemur* genome and obtained total 21 contigs (1.74 Mb) as likely bacterial contigs (Table 2, Fig 1A). Remarkably, among these, one long contig (contig55, 1.41 Mb) showed a high-level mapping rate with *Wolbachia* genomes (98.8%), suggesting this contig might be a partial genome of the symbiotic *Wolbachia* bacteria in *G. flavifemur*. In addition, we found 17 relatively short contigs (0.3 Mb in total) with best hits against *Arsenophonus nasoniae*, a symbiont that causes male killing in *Nasonia vitripennis* [26], suggesting this symbiont may also infect *G. flavifemur*. The last three contigs (0.03 Mb in total) contained fragments from *Ignisphaera aggregans* (archaeal species firstly isolated from a near neutral, boiling spring) or *Methanobrevibacter smithii* (the main human methanogen almost always found in the digestive tract of adults) were identified as contaminating bacteria.

We next analyzed the *Wolbachia* coting (*wGfla*) we found above. First, 1,532 protein coding genes were predicted in this contig by RAST. Phylogenetic analysis of genome-wide single-copy genes of insect-associated *wolbachia* genomes (36 genomes) indicated that the *wGfla* was closed to *wDacB*, and belonged to supergroup B (Fig 1). Whole genome alignment analysis between *wGfla* and *wDacB* showed a pattern of genome synteny (Fig 2).

Table 1. *Wolbachia* genome used for phylogenetic analysis.

| Proteome ID | Organism | Host Family | Refseq ID |
| --- | --- | --- | --- |
| UP000297624 | *Wolbachia* endosymbiont of *Drosophila mauritiana* | Diptera | GCF_004685025.1 |
| UP000501527 | *Wolbachia* endosymbiont of *Diaphorina citri* | Diptera | GCF_013096725.2 |
| UP000013003 | *Wolbachia* endosymbiont of *Drosophila simulans* wHa (Strain: wHa) | Diptera | GCF_000376605.1 |
| UP000311529 | *Wolbachia* endosymbiont of *Leptopilina clavipes* (Strain: GBW) | Hymenoptera | GCF_006334525.1 |
| UP000008814 | *Wolbachia pipientis* subsp. *Culex pipiens* (strain wPip) | Diptera | GCF_000073005.1 |
| UP000306512 | *Wolbachia* endosymbiont of *Drosophila yakuba* (Strain: wYak_CY17C) | Diptera | GCF_005862115.1 |
| UP000324141 | *Wolbachia* endosymbiont of *Chrysomya megacephala* | Diptera | GCF_008245065.1 |
| UP000031663 | *Wolbachia* endosymbiont of *Cimex lectularius* (Strain: wCle) | Hemiptera | GCF_000829315.1 |
| UP000078016 | *Wolbachia* endosymbiont of *Dactylopius coccus* (Strain: wDacB) | Hemiptera | GCA_001648015.1 |
| UP000309942 | *Wolbachia* endosymbiont of *Aedes albopictus* | Diptera | GCF_004795415.1 |
| UP000001293 | *Wolbachia* sp. subsp. *Drosophila simulans* (strain wRi) | Diptera | GCF_000022285.1 |
| UP000195810 | *Wolbachia* endosymbiont wPip_Mol of *Culex molestus* (Strain: wPip_Mol) | Diptera | GCF_000723225.2 |
| UP000315509 | *Wolbachia* endosymbiont of *Carposina sasakii* | Lepidoptera | GCF_006542295.1 |
| UP000321156 | *Wolbachia* endosymbiont of *Drosophila ananassae* (Strain: W2.1) | Diptera | GCF_008033215.1 |
| UP000025842 | *Wolbachia* endosymbiont of *Glossina morsitans* morsitans | Diptera | GCF_000689175.1 |
| UP000077048 | *Wolbachia* endosymbiont of *Laodelphax striatellus* (Strain: wStri) | Hemiptera | GCF_001637495.1 |
| UP000004878 | *Wolbachia* endosymbiont of *Muscidifurax uniraptor* (Strain: wUni) | Hymenoptera | GCF_000174095.1 |
| UP000282698 | *Wolbachia* endosymbiont of *Bemisia tabaci* (Strain: China 1) | Hemiptera | GCF_003999585.1 |
| UP000077579 | *Wolbachia* endosymbiont of *Dactylopius coccus* (Strain: wDacA) | Hemiptera | GCA_001648025.1 |
| UP000004540 | *Wolbachia* endosymbiont of *Culex quinquefasciatus* JHB (Strain: JHB) | Diptera | GCF_000156735.1 |
| UP000013004 | *Wolbachia* endosymbiont of *Drosophila simulans* wNo (Strain: wNo) | Diptera | GCF_000376585.1 |
| UP000218099 | *Wolbachia* endosymbiont of *Drosophila subpulchrella* (Strain: wSpc) | Diptera | GCF_002300525.1 |
| UP000284695 | *Wolbachia* endosymbiont of *Drosophila ananassae* (Strain: wAna_India) | Diptera | GCF_003671365.1 |
| UP000502750 | *Wolbachia* endosymbiont of *Diaphorina citri* | Diptera | GCF_013096535.2 |
| UP000296807 | *Wolbachia* endosymbiont of *Drosophila mauritiana* (Strain: wMau) | Diptera | GCF_004795955.1 |
| UP000095451 | *Wolbachia* endosymbiont of *Drosophila incompta* (Strain: wInc_Cu) | Diptera | GCF_001758565.1 |
| UP000296800 | *Wolbachia* endosymbiont of *Drosophila mauritiana* (Strain: wMau) | Diptera | GCF_004795975.1 |
| UP000284402 | *Wolbachia* endosymbiont of *Drosophila ananassae* (Strain: wAna_Indonesia) | Diptera | GCF_003671375.1 |
| UP000501641 | *Wolbachia* endosymbiont of *Diaphorina citri* | Diptera | GCF_013096355.2 |
| UP000237178 | *Wolbachia* sp. subsp. *Drosophila simulans* (strain wRi) | Diptera | GCF_002907405.1 |
| UP000305510 | *Wolbachia* endosymbiont of *Drosophila santomea* (Strain: wSan_Quija630.39) | Diptera | GCF_005862095.1 |
| UP000306265 | *Wolbachia* endosymbiont of *Drosophila teissieri* (Strain: wTei_cascade_4_2) | Diptera | GCF_005862135.1 |
| UP000284289 | *Wolbachia* endosymbiont of *Drosophila ananassae* (Strain: wAna_Hawaii) | Diptera | GCF_003671405.1 |
| UP000296278 | *Wolbachia* endosymbiont of *Brugia malayi* | Nematoda | GCF_004795935.1 |
| UP000001942 | *Neorickettsia sennetsu* (strain ATCC VR-367 / Miyayama) (*Ehrlichia sennetsu*) | Outgroup | GCF_000013165.1 |
| UP000001943 | *Anaplasma phagocytophilum* (strain HZ) | Outgroup | GCF_000013125.1 |
| UP000033562 | *Candidatus Neoehrlichia* lotoris (Strain: RAC413) | Outgroup | GCF_000964795.1 |
| UP000007307 | *Anaplasma marginale* (strain Florida) | Outgroup | GCF_000020305.1 |

Table 2. Bacterial contigs identified in *G. flavifemur* genome assembly.

| Contig | Contig Length | Bacterial Length | Hits units | Proportion of hits units | Best hits |
| --- | --- | --- | --- | --- | --- |
| Contig427 | 8909 | 6960 | 9 | 1 | *Arsenophonus nasoniae* |
| Contig284 | 21964 | 14004 | 22 | 1 | *Arsenophonus nasoniae* |
| Contig430 | 8435 | 7826 | 9 | 1 | *Arsenophonus nasoniae* |
| Contig358 | 14274 | 12353 | 15 | 1 | *Arsenophonus nasoniae* |
| Contig448 | 7157 | 7105 | 8 | 1 | *Arsenophonus nasoniae* |
| Contig425 | 9183 | 5543 | 10 | 1 | *Arsenophonus nasoniae* |
| Contig344 | 15333 | 11645 | 16 | 1 | *Arsenophonus nasoniae* |
| Contig450 | 7132 | 5905 | 8 | 1 | *Arsenophonus nasoniae* |
| Contig268 | 25414 | 25356 | 26 | 1 | *Arsenophonus nasoniae* |
| Contig469 | 6101 | 5252 | 7 | 1 | *Arsenophonus nasoniae* |
| Contig466 | 6155 | 6121 | 7 | 1 | *Arsenophonus nasoniae* |
| Contig55 | 1408188 | 897570 | 1392 | 0.9879 | *Wolbachia* endosymbiont |
| Contig301 | 20027 | 19634 | 20 | 0.9524 | *Arsenophonus nasoniae* |
| Contig168 | 56106 | 29206 | 54 | 0.9474 | *Arsenophonus nasoniae* |
| Contig257 | 26762 | 13504 | 25 | 0.9259 | *Arsenophonus nasoniae* |
| Contig175 | 51574 | 22067 | 46 | 0.8846 | *Arsenophonus nasoniae* |
| Contig451 | 7110 | 4836 | 7 | 0.875 | *Arsenophonus nasoniae* |
| Contig398 | 11091 | 2798 | 10 | 0.8333 | *Arsenophonus nasoniae* |
| Contig378 | 12362 | 979 | 7 | 0.5385 | *Methanobrevibacter smithii* |
| Contig349 | 14634 | 1072 | 7 | 0.4667 | *Methanobrevibacter smithii* |
| Contig417 | 9399 | 462 | 4 | 0.4 | *Ignisphaera aggregans* |


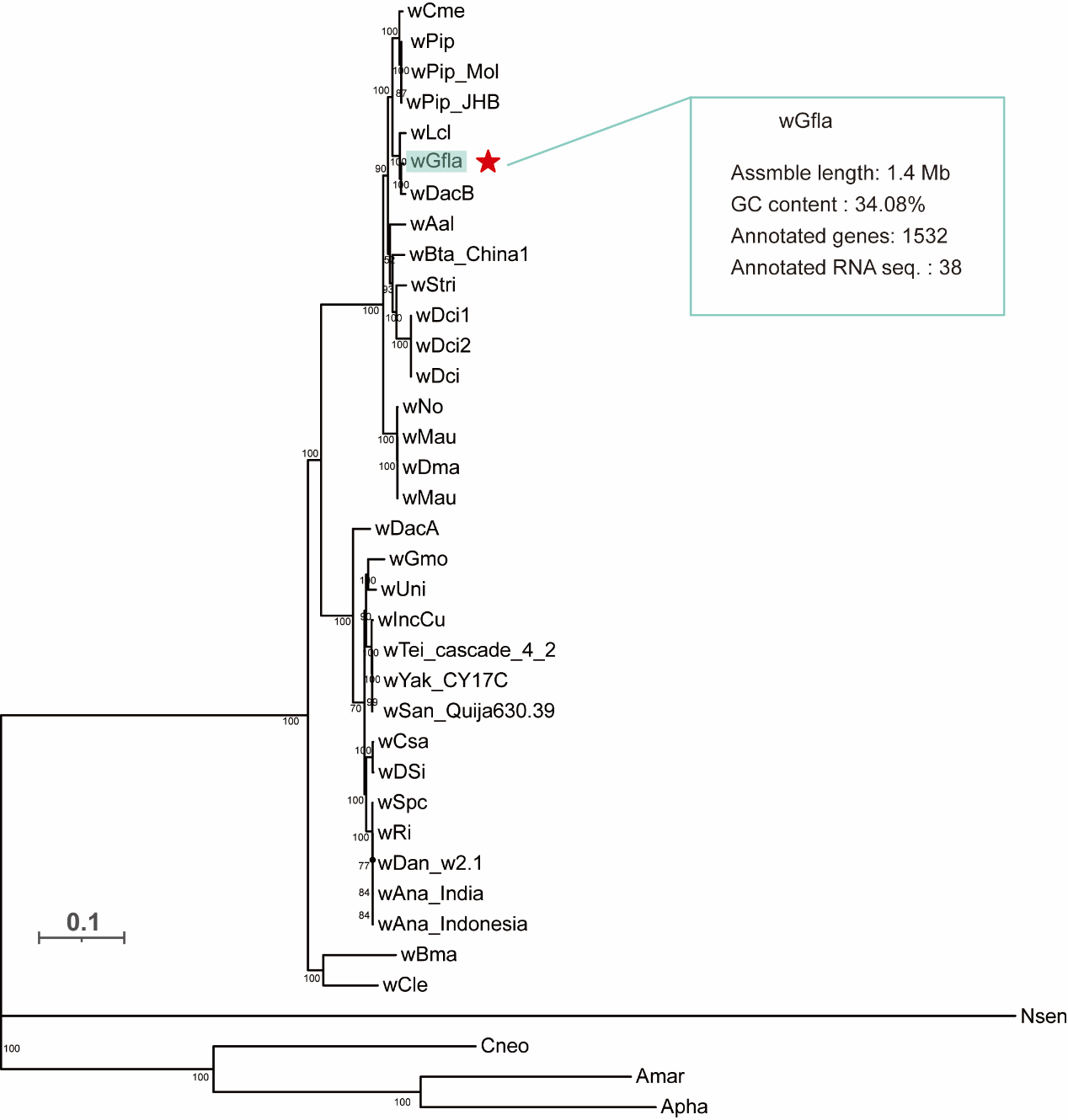


Fig 1. Phylogenetic analysis of *wGfla*. Phylogenetic analysis of *wGfla.* The maximum likelihood tree was built according to 192 single copy genes using IQ-TREE v2.1.2 with the JTT + F + R4 model. Bootstrap values are indicated at the respective nodes (only values > 50% are shown).


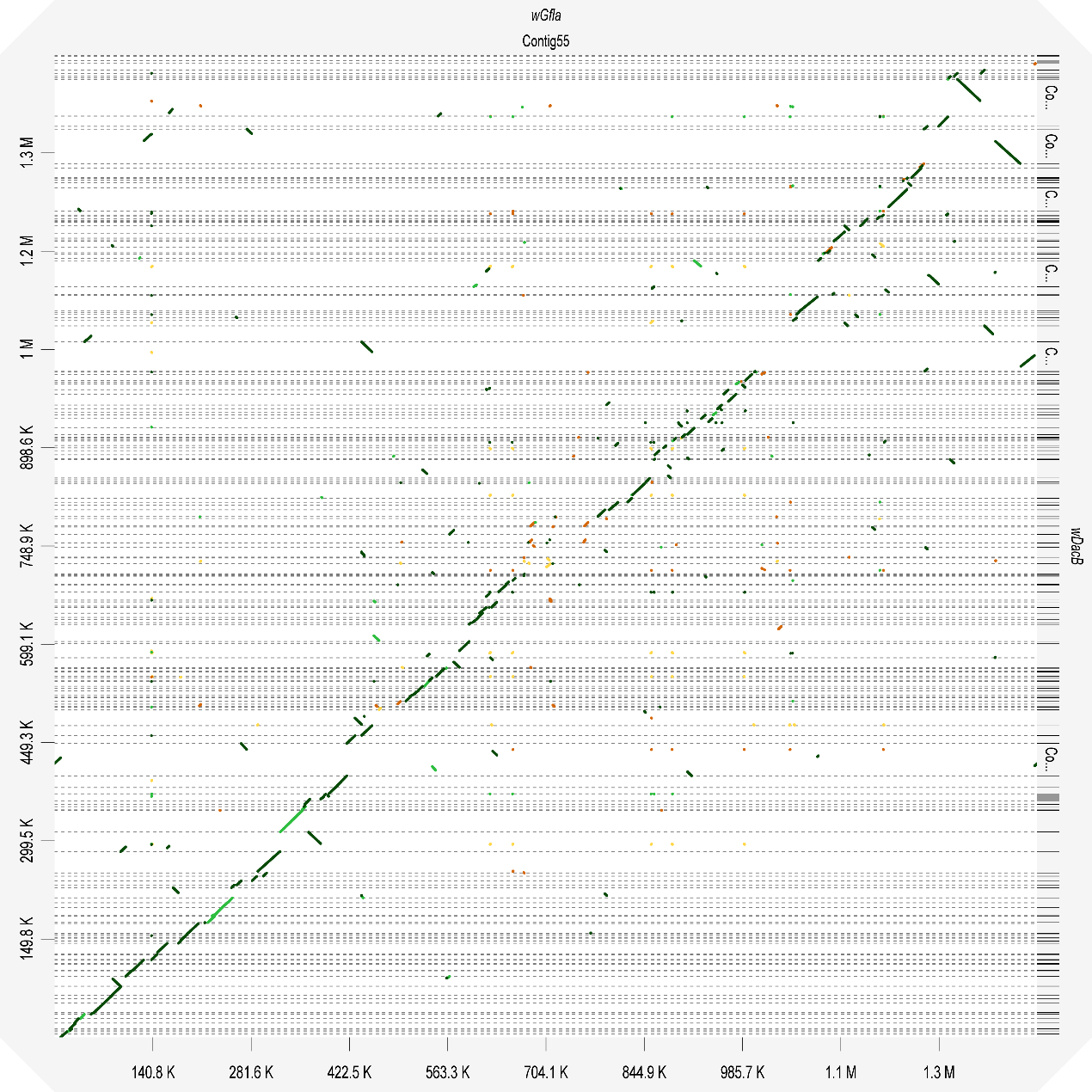


Fig 2. Whole genome alignment of *wGfla* and *wDacB*. The whole genome alignment was performed using the online service for comparative genomic analysis, D-GENES.
